# Principles of computer-controlled linear motion applied to an open-source affordable liquid handler for automated micropipetting

**DOI:** 10.1038/s41598-020-70465-5

**Published:** 2020-08-12

**Authors:** David C. Florian, Mateusz Odziomek, Cerie L. Ock, Hannah Chen, Scott A. Guelcher

**Affiliations:** 1 Department of Biomedical Engineering, Vanderbilt University, Nashville, TN 37235 USA; 2 Center for Bone Biology, Vanderbilt University Medical Center, Nashville, TN 37232 USA; 3 Department of Chemical and Biomolecular Engineering, Vanderbilt University, Nashville, TN 37235 USA

**Keywords:** Biomedical engineering, Mechanical engineering

## Abstract

OTTO is an open-source automated liquid handler that can be fabricated at a cost of $1,500 using off-the-shelf and 3D-printable parts as an alternative to commercial devices. Open-source approaches have been applied to build syringe pumps, centrifuges, and other laboratory equipment. These devices are affordable but generally rely on a single motor to perform simple operations and thus do not fully utilize the potential of the Maker Movement. Open-source linear actuators and microcontrollers enable the fabrication of more complex laboratory instruments that rely on 3D positioning and accurate dispensing of fluids, such as automated liquid handlers. These instruments can be built rapidly and affordably, thereby providing access to highly reproducible sample preparation for common biological assays such as qPCR. We applied the design principles of speed and accuracy, unattended automation, and open-source components to build an automated liquid handler that controls micropipetting of liquids in 3D space at speeds and positional resolutions required for qPCR. In benchmarking studies, OTTO showed accuracy and sample preparation times comparable to manual qPCR. The ability to control linear motion and liquid dispensing using affordable off-the-shelf and 3D-printable parts can facilitate the adoption of open-source automated liquid handlers for qPCR, bioplotting, and other bioinstrumentation applications.

## Introduction

At the onset of a viral outbreak, quantitative polymerase chain reaction (qPCR) tests are commonly employed to screen patients for disease^1^. Compared to serology tests, which search for antibodies that are reactive to the virus, qPCR-based tests are more sensitive and can be implemented faster but have longer turnaround times^2–5^. During the COVID-19 outbreak, health care officials promptly recruited technicians to run qPCR on samples from patients who experienced an exposure event to detect the presence of viral RNA^6^. However, preparing samples for qPCR is prone to human error and time consuming^7–9^, resulting in decreased reproducibility and increased costs^10–12^. These limitations have been mitigated in part by robotic liquid handlers that are more precise and faster than their human counterparts. However, commercial liquid handlers are expensive systems with recurring maintenance contracts that the majority of laboratories cannot afford^13^. The rarity of these instruments may have contributed in part to hospitals being overwhelmed by the large number of patient samples during the COVID-19 pandemic.

The Maker Movement is an educational initiative that focuses on the innovative application of open-source technologies to solve problems at multiple scales. This movement was enabled by the advent of additive manufacturing (i.e., 3D printing), a process for converting a digital model into a physical part that provides affordable access to digital fabrication. The Maker Community has a history of generating solutions for public health problems, such as the design of low-cost and readily manufacturable ventilators and 3D-printable face shields during the COVID-19 pandemic^14^. Academic research groups have also embraced this movement by building do-it-yourself (DIY) affordable versions of laboratory instruments. Examples of DIY lab equipment include syringe pumps^15–17^, plate readers^18^, and bioplotters^19,20^. The designs and code for many of these DIY instruments have been made freely available online for others to replicate and modify. These open-sourced tools have the potential to enhance the productivity of laboratories if they can be implemented at a reasonable cost and effort.

Automated DIY liquid handlers have been reported for cell culture^21^, droplet formation^15^, and other applications. However, these bioinstruments do not provide sufficient volumetric and positional accuracy and resolution for qPCR applications. Motivated by the COVID-19 pandemic and the Maker Movement, we designed an open-source liquid handler, known as OTTO, that can automatically prepare samples for qPCR. OTTO costs approximately $1,500 and works with most commercially available micropipettes and plastic labware (e.g., pipette tips, well plates, and microcentrifuge tubes), thereby eliminating the need to purchase custom materials. Furthermore, OTTO can be built with a small number of commonly available tools (e.g., hex keys and wire cutters). All the resources required to build OTTO are available online (https://OpenLiquidHandler.com), with the assembly instructions tailored to personnel with minimal experience in mechatronics.

Herein we highlight the features of OTTO that make it a robust, accurate, and reliable automated liquid handling system for generating reproducible qPCR data. The low cost and readily available components allow for a system like OTTO to be mass-produced during a pandemic for rapid sample testing. Once the crisis has subsided, OTTO can be repurposed for other laboratory assays or disassembled and sold to recover costs.

## Methods

### Overview of systems

The design of automated Computer Numerical Controlled (CNC) machines can be divided into three systems: mechanical, electrical, and software. In high-end CNC machines, these systems become increasingly integrated and complex in order to accomplish sophisticated tasks. OTTO’s systems are designed to be flexible and discrete, thereby decreasing complexity and allowing users to substitute components to meet the user’s needs.

### Mechanical description

**Figure 1:**
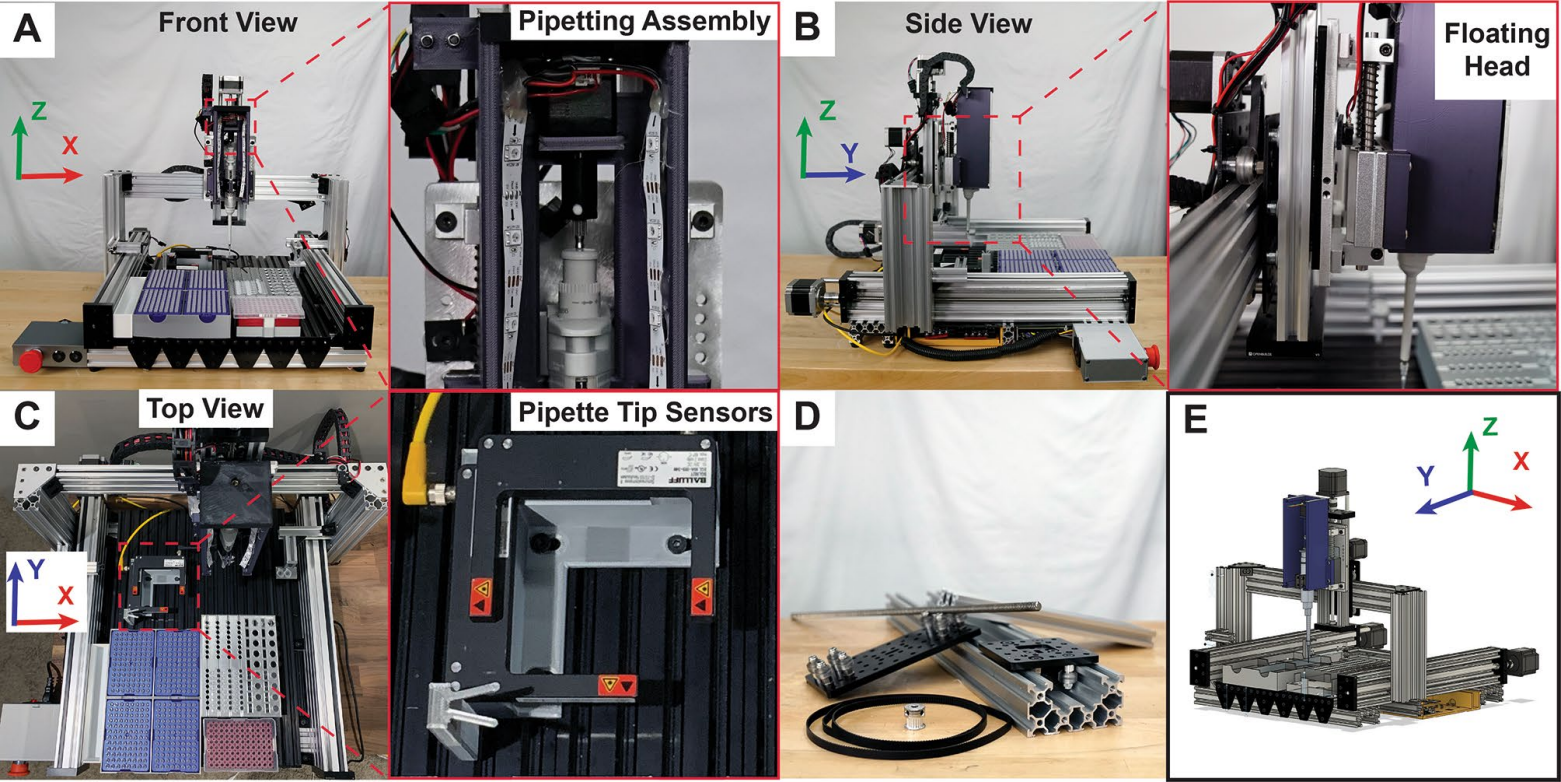
Photographs and 3D reconstructions illustrating the key features of OTTO. (**A**) Front view of the X- and Z-axes and pipetting assembly. (**B**) Side view of the Y- and Z-axes and floating head. (**C**) Top view of the work envelope (X- and Y-axes) and pipette tip sensors. (**D**) Open-source rails, carriages, and transmissions from OpenBuilds used to construct OTTO’s linear actuators. (**E**) 3D renderings of OTTO based on a CAD model.

OTTO is a 3D linear motion platform constructed from open-source linear rails and carriages (OpenBuilds, Monroeville, NJ) that is capable of positioning a manual micropipette anywhere within its work envelope (Fig. 1A,D) (openbuildspartstore.com/). A linear actuator sits above the micropipette, forming the pipetting assembly (Fig. 1A), and automates the process of pressing and depressing the plunger on the micropipette. To regulate the force when loading a new tip onto the micropipette, the pipetting assembly rides on a pair of vertical, spring-loaded linear rods, creating the floating head assembly (Fig. 1B). The tip loading pressure can be increased or decreased using springs with higher or lower spring constants, respectively. All of the accessories required for an experiment (e.g., tip boxes, well plates, etc.) are attached to the bottom of the work envelope with 3D-printed holders and fasteners (Fig. 1C, see Supplementary Figure S1 online). The part numbers, quantities, and (where appropriate) Computer Aided Design (CAD) models (Fig. 1E) for the mechanical components are available online at https://OpenLiquidHandler.com/Mechanical and can be found as Supplementary Tables S1 and S2.

### Electrical description

**Figure 2:**
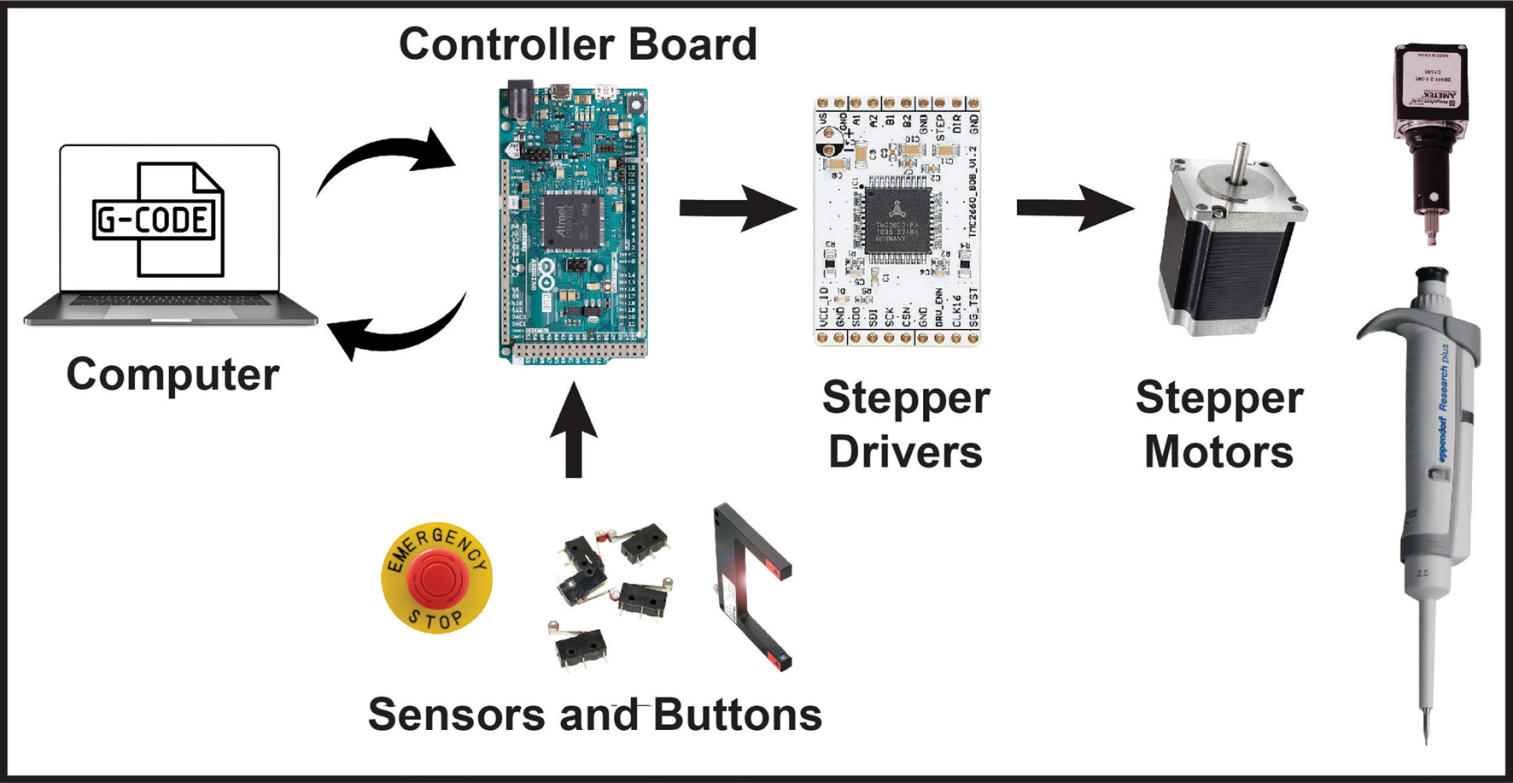
Simplified electrical schematic of OTTO. An Arduino-based controller board coordinates input from sensors and output to stepper drivers according to G-code sent over a serial connection from a computer.

Bipolar hybrid stepper motors move the timing belt (X-axis) and lead screws (Y- and Z-axes) and provide the rotary power for the linear actuator in the pipetting assembly. TMC-2660 stepper drivers (Trinamic, Hamburg, Germany) coordinate the rotation of each stepper motor based on step and direction signals sent from a 32-bit Arduino Due (Arduino LLC, Ivrea, Italy, see Supplementary Figure S2). The Arduino Due receives high-level input from a computer via USB serial connection (Fig. 2). Mechanical limit switches found at the maxima of each axis establish a repeatable coordinate system and allow for a common home position to be found. Two through-beam sensors (Balluff, Florence, KY) located within the work envelope are used to check the concentricity of the micropipette’s tip holder compared to its barrel and to detect the presence of pipette tips (Fig. 1C). Mechanical switches can be a substitute for the Balluff through-beam sensors for further cost savings as seen in Supplementary Figure S3. The motors, limit switches, and through-beam sensors are powered by a 350 W 24 V regulated DC power supply. The 24 V output signals of the limit switches and through-beam sensors are converted to the Arduino Due’s digital pins through optocouplers (see Supplementary Figure S4 and Supplementary Table S3). A wiring diagram for all the electrical systems with part numbers is provided online (https://OpenLiquidHandler.com/Electrical).

### Software description

Programming the automatic liquid handler is accomplished using a Python 3 application that features a Tkinter graphical user interface (GUI) (see Supplementary Figures S5–S9). The user selects the assay to run and inputs the geometry and location of the plastic labware within the build envelope. The program then generates G-code in accordance with the ISO 6,983 standard (see Supplementary Table S4), which is fed line-by-line to the Arduino Due. Between lines, the computer awaits status updates from the Arduino and adjusts subsequent G-code if errors are reported. This program has been developed to run on Windows. The Arduino Due runs a custom firmware that functions as a simplified G-code interpreter and was programmed in the Arduino Integrated Development Environment (IDE). The Arduino Due relies on the AccelStepper library to convert positional coordinates into step and direction pulses to move the stepper motors according to predefined acceleration and velocity curves. Both the Arduino and Python code are maintained online (https://OpenLiquidHandler.com/Software).

## Results

The technical criteria that guided the design of OTTO were established from the constraints of running a qPCR test in the laboratory. Thus, reproducible qPCR sample preparation was critical for the success of our design. The objective was to create an approachable design consistent with Maker Movement principles by selecting user-friendly components to accommodate potential users who are unfamiliar with CNC machines. We have also found that these design principles apply more generally to other biological assays that rely on 3D automated liquid handling.

### Speed and accuracy

During qPCR sample preparation, technicians combine reagents from microcentrifuge tubes into a well plate, which requires switching pipette tips between reagents. An experienced technician can prepare the standard curves and samples that fill a 96-well plate in 30–45 min. Since the utility of a CNC machine is assessed by how fast it can perform a manual task, an important goal was to automate the process of sample preparation without increasing its duration. Due to the need to switch tips between pipetting reagents to prevent cross-contamination, a micropipette spends more time moving to and from the tip box than it does dispensing liquid. Therefore, the velocities at which the linear actuators are able to move the micropipette in the X-, Y-, and Z-directions are important variables to maximize.

**Figure 3:**
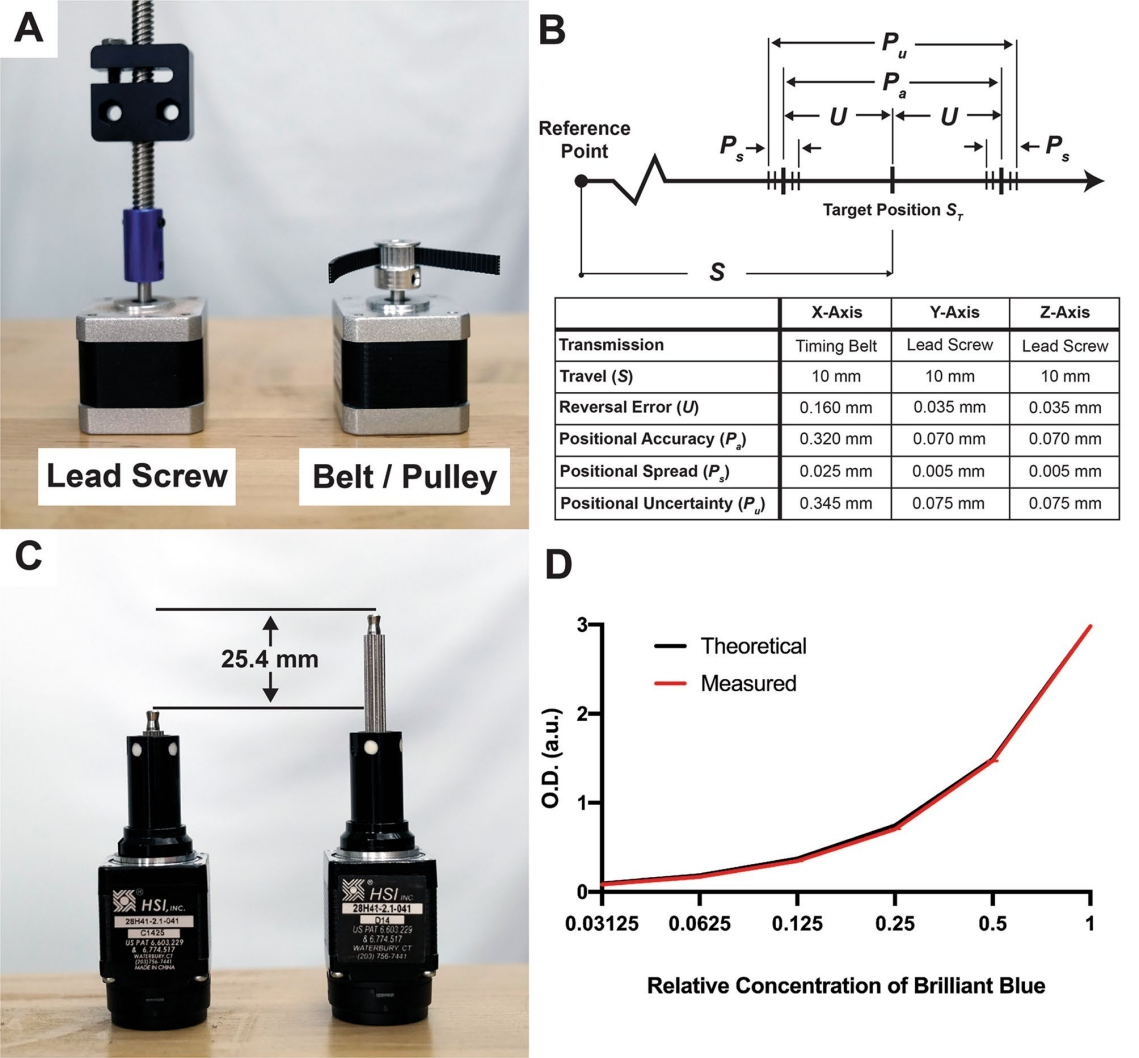
Positional and pipetting accuracy of OTTO. (**A**) Photograph of the two types of transmissions used to convert the rotation of stepper motors into linear movement: lead screw and pulley with timing belt. (**B**) A schematic illustrating how reversal error (*U*) and variances in positioning (*P*_*s*_) affect positional accuracy (*P*_*A*_) and positional uncertainty (*P*_*u*_). These parameters were assessed for OTTO after moving 10 mm in opposite directions. A Clockwise Tools DITR-0055 Electronic Digital Indicator (0.001 mm resolution) was used to measure the distance traveled by each axis (*n* = 10) (**C**) Haydon Kerk captive linear stepper motors with 25.4 mm stroke length. (**D**) Optical density (O.D.) measured at 625 nm of a 1:2 serial dilution of Brilliant Blue FCF #1 dye to illustrate the pipetting accuracy of OTTO (*n* = 4).

The maximum speed of a linear actuator is determined by how fast the motor rotates and by the linear distance the actuator travels per rotation of the motor shaft. We selected standard bipolar hybrid stepper motors for our linear actuators because they are inexpensive and capable of precise movements without complex positional feedback loops. However, the tradeoff for their simplicity is a dramatic reduction in torque with increasing rotations per minute (RPM)^22^. Consequently, stepper motors are typically operated at less than 1,000 RPM. These lower speeds can be offset by choosing linear motion components that move farther per rotation of the motor’s shaft. Screws and timing pulleys (Fig. 3A) are common types of transmissions that convert the stepper motor rotation into linear movement. Increasing the length of the lead on a screw or the diameter of a pulley will increase the distance the actuator moves per rotation of the motor, but this increase in speed results in a proportional decrease in positional resolution since stepper motors take discrete steps.

We aimed to identify the minimum positional resolution necessary for liquid handling to maximize speed. Interestingly, we found that the process requiring the highest positional resolution was loading a pipette tip. We sampled a variety of tips from commercial vendors and found that positional errors as small as 500 μm could result in the micropipette failing to load the tip. Not all tips required this level of positional resolution, but small (< 20-μL) tips that are commonly used in qPCR preparation required 500-μm resolution due to their size. This positional resolution requirement is within the capabilities of a timing pulley/belt transmission, which we employed for the X-axis. We were able to achieve a motor step size of 0.1125 degrees through a technology known as microstepping that is available on most commercial stepper drivers^23^. Combining this small step size with a 20-tooth pulley and belting with a 2-mm pitch, the X-axis is able to obtain a theoretical resolution of 12.5 μm. While this resolution is over an order of a magnitude greater than required, reversal error (*U*), which is a combination of backlash and hysteresis^24^, limits the positional accuracy (*P*_*A*_) of this linear actuator to 320 μm (Fig. 3B). This reversal error was relatively consistent with a low positional spread (*P*_s_) and could be compensated for digitally if higher resolutions were needed.

We were able to obtain a maximum speed of 200 mm/s because the Arduino Due microcontroller can generate up to 16,000 step pulses per second when running the AccelStepper Arduino library (16,000 steps/s × 12.5 μm/step)^25^. At this maximum speed the stepper motor rotates at 300 RPM, at which conditions it has the highest amount of torque. In practice, applying a conservative acceleration of 100 mm/s allows the X-axis to transport the micropipette across its full 300 mm of travel in 3.5 s. Increasing the acceleration and/or pulley size would decrease this time of flight, but these adjustments could also lead to increased inaccuracies in the movement due to torque constraints.

In contrast to the X-axis linear actuator, we used 8-mm lead screws for the Y- and Z-axes (Fig. 3A). Lead screws are typically slower than a timing belt/pulley configuration but have the benefit of increased stability and accuracy. For example, the friction between a lead screw and its nut prevents gravity from pulling the Z-actuator downward, which would cause the micropipette to impact the bottom of the machine when the power is switched off. Furthermore, OTTO utilizes a gantry-style linear motion configuration, where the workspace remains stationary as the micropipette moves in all three degrees of freedom. This configuration requires the Y-actuators to bear the weight of both the X- and Z-actuators. Lead screws are designed to transfer large loads without jamming or stretching, a common problem with timing belts^26^. In our setup, these lead screw-based actuators can obtain a maximum speed of 50 mm/s.

This linear motion configuration has proven to be reliable and reproducible. However, due to the discrepancy in speeds between the X and Y linear actuators, we have designed the workspace to minimize travel in the Y-axis. The pipette tip remover and two holders for commonly used reagents, such as water and PCR mastermix, are carried alongside the pipette and are accessible through moving only the X actuator. These motion planning techniques allow OTTO to load a tip, move to a microcentrifuge tube, mix and draw the reagent into the tip, move to another tube or well, dispense the reagent, and remove the tip in approximately 35 s. The time required to perform this unit operation can be used to predict the duration of sample preparation if OTTO mimics manual liquid handling techniques. However, with computer control over the micropipette, OTTO can divide a volume within a single pipette tip between multiple wells when reagents, like mastermix, remain constant across multiple wells. This time-saving technique has enabled OTTO to prepare qPCR reactions that fill a 96-well plate in less than an hour.

The accurate and precise dispensing of fluids at the μL-scale is critical for reliable and reproducible qPCR data. However, building a custom motorized micropipette is expensive and requires special tools. We decided to automate the process of depressing the plunger of a manual micropipette, which is simpler than designing a motorized pipette and allows labs to repurpose available pipettes. Furthermore, when paired with a linear actuator with a sufficiently high positional resolution, the pipetting accuracy will be dictated by that of the manual pipette. For the pipetting actuator, we selected a Haydon Kerk (Waterbury, CT) captive linear stepper motor in which an integrated threaded drive screw directly actuates a shaft (Fig. 3C). This low-profile and lightweight linear actuator is capable of a 25.4-mm stroke length, which is larger than the travel distance of most commercial micropipette plungers. The actuator moves 25.4 μm per step, which can be decreased further by microstepping. To test the accuracy of the automated pipetting, a 1:2 serial dilution was performed with a solution of Brilliant Blue FCF #1 dye in distilled water (Fig. 3D). OTTO used an Eppendorf Research 2,100 10-μL pipette to dispense 5 μL of water in a strip of PCR tubes before serial diluting a stock solution of dye. Absorbance readings showed an average pipetting error of 2.5%, which is comparable to the error reported in the micropipette specification (1.5%)^27^.

### Unattended automation

DIY machines are commonly perceived to be less reliable than their commercial counterparts. A core design constraint for OTTO was that it should not fail due to a source of unknown error. There are many factors that can invalidate the results of a qPCR test, from using improper primers to issues with cDNA generation, which can take hours to identify. OTTO was designed to eliminate liquid handling malfunctions that would lead to low adoption of this technology, since users would not have confidence to leave OTTO unattended. One of the largest benefits from automating qPCR preparation is the ability to set up the liquid handler in a cold room to prepare these temperature- and light-sensitive assays without human intervention. Therefore, we have implemented error correcting and reporting mechanisms that allow OTTO to adapt to problems and notify the user when an error occurs.

**Figure 4:**
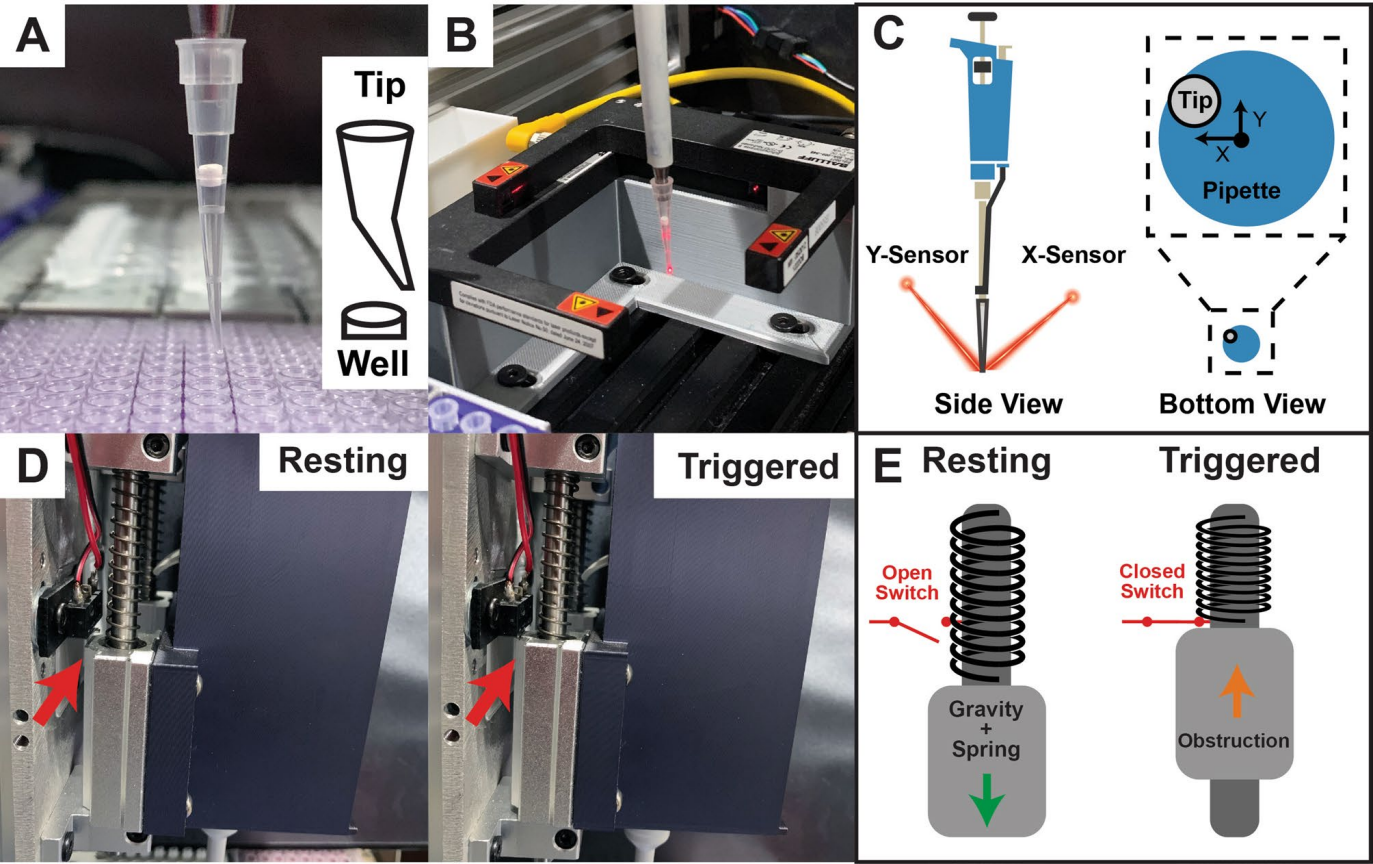
Error sensing features of OTTO. (**A**) Photograph demonstrating how an eccentric (i.e., bent) pipette tip can miss the desired well, causing erroneous results. (**B**) Photograph of a pipette sensing module that uses two Balluff through-beam sensors to compare the location of the micropipette barrel with the bottom of the pipette tip. (**C**) Schematic illustrating how an X- and Y-coordinate offset can compensate for a misaligned tip. (**D**) Photographs of the Z-axis floating head before and after meeting an obstruction, which triggers the mechanical limit switch (red arrow). (**E**) Schematic of the floating head’s operating principle. The force of the spring, as well as gravity, prevents the mechanical limit switch from being tripped during normal operation. However, an obstruction in the Z-axis will force the micropipette upwards, triggering the mechanical limit switch and notifying the microcontroller that there is an obstruction.

In initial prototypes, we found that the most significant variable was the shape of the pipette tips. A small percentage (< 1%) of tips were malformed, which caused the dispensing end of the pipette tip to not be aligned with the micropipette (Fig. 4A). Therefore, early versions of OTTO could miss a well or tube when collecting or dispensing liquid. To address this limitation, two Balluff transparency sensors, one aligned with the X-axis and one with the Y-axis, were used to measure the concentricity of the tips with the micropipette (Fig. 4B). A coordinate offset was applied to compensate for any bent tips (Fig. 4C). These two sensors ensure that tips are properly loaded and ejected, thereby preventing cross-contamination between samples. While checking the alignment of each tip is time consuming, in many cases doubling the time it takes OTTO to run an assay, this error checking can prevent costly mistakes.

Another source of error is failure to load a pipette tip, which in our testing occurred when the tip was improperly seated in its holder. Failure to load the tip results in the absence of reagents in certain wells or tubes during qPCR preparation. This error had further implications because ill-positioned pipette tips could obstruct the movement of the micropipette during the tip loading process. Without any feedback when this obstacle is detected, the Z-axis would continue to lower but the movement would be translated into bending the shaft of the micropipette, resulting in loss of alignment with the calibrated coordinate system. There are other scenarios where it would be important for the liquid handler to sense obstructions in the Z-direction, such as a closed microcentrifuge tube. To address these potential sources of error, we implemented a floating head design, inspired by plasma cutters, that provides feedback when a tool such as a micropipette encounters an obstacle in the Z-direction (Fig. 4D). We incorporated springs in the floating head to allow for sufficient force for the micropipette to load a tip without triggering the obstruction sensor at the top of the assembly (Fig. 4E). This modification improved the reliability of OTTO because the software was programmed to retract the Z-axis whenever the obstruction sensor was triggered and inform the user.

### Open-source and modular components

A significant limitation of proprietary parts is the lack of accessible information on their functionality and programming, which prevents users from modifying or repairing the machine and creates a barrier to adoption. We aimed to eliminate these obstacles by choosing open-source components that are clearly documented and popular in the Maker Community. For example, we used an Arduino Due as OTTO’s microcontroller, which is based on the well-established Arduino platform (https://www.arduino.cc/). The Arduino ecosystem serves all levels of user experience through tutorials, supplemental information, and widely used forums. By selecting common parts such as the Arduino, users can deploy components they already own, further reducing the cost and learning curve involved with building this machine. As for the specialized parts that are required for OTTO’s functionality, such as the micropipette holder, we provide 3D designs that are fully customizable, allowing users to generate and 3D-print holders that fit their micropipettes and disposable plasticware (Supplementary Table S2).

Multiple micropipettes with different working ranges of volumes are often required to complete an assay. However, designing OTTO to automatically switch between micropipettes would have significantly increased its complexity. Alternatively, we implemented a modular design that allows the entire micropipette assembly to be replaced. This modularity also allows for the micropipettes to be exchanged for syringe pumps to handle large volumes of liquid or to extrude gels. Other components, such as the rails, are also readily interchangeable for different size work envelopes. Thus, modifications and expansions can be easily done without compromising the basic functionality of the machine.

The modular and open-source design approach extends to OTTO’s software as well. Instead of repurposing existing software to coordinate the linear motion, like 3D printer firmwares, we designed a simplified firmware that runs OTTO with no superfluous features (see Supplementary Table S4). This makes the code less overwhelming and easily modifiable to users who are unfamiliar with computer programming. However, for those who want to use their own electronics to control OTTO, the Python 3 GUI that assists users in setting up experiments is compatible with any controller board that accepts G-code via serial communication.

### Benchmarking

OTTO has been successfully deployed to prepare samples for either of the two common quantification methods for qPCR: Relative Standard Curve (RSC) or Comparative Cycle Threshold (ΔΔCT). When preparing samples for RSC quantification, OTTO generates a serial dilution of the cDNA prior to pipetting the reagents and samples into the assay plate. This serial dilution is the basis for the standard curve that samples will be compared to for absolute quantification. With ΔΔCT sample preparation, OTTO does not generate a standard curve and the samples are compared to a control for relative quantification.

**Figure 5:**
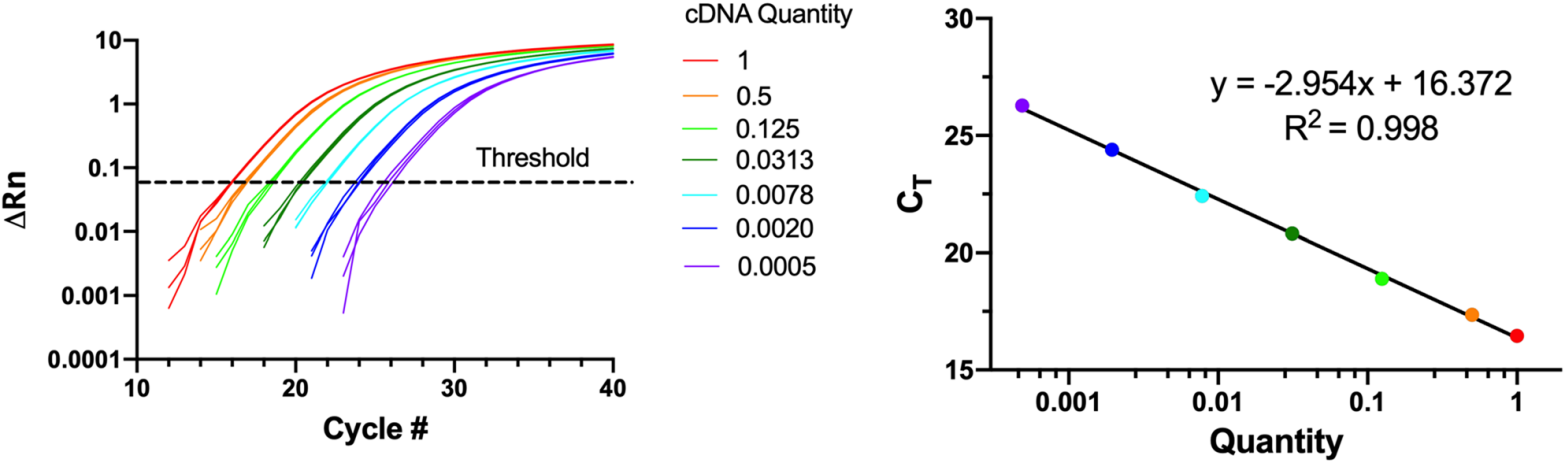
qPCR results after automatic sample preparation by OTTO. (**A**) cDNA generated from in vitro hMSC cultures by reverse transcriptase was automatically diluted first 1:2 and then subsequently serial diluted 1:4 to form the basis of a standard curve. Diluted cDNA was added in triplicates to a 96-well plate before TaqMan mastermix and primers for the 18S housekeeping gene were added to each well. An Applied Biosystems 7,500 Real-Time PCR System amplified the product for 40 cycles. ΔRn is a normalized reporter value for the fluorescent signal coming from the PCR product after each cycle of amplification minus the baseline signal generated from the instrument. (**B**) The quantity of cDNA was plotted against the cycle number at which each product exceeded background fluorescent levels (C_T_). The resulting standard curve had an R^2^ value of 0.998.

As a benchmarking test, OTTO prepared samples for a RSC qPCR reaction using TaqMan mastermix and primers for the 18S housekeeping gene. mRNA was isolated from in vitro cultures of human Mesenchymal Stem Cells (hMSCs) and converted to cDNA by a reverse transcriptase reaction. The undiluted cDNA, nuclease-free water, primers, mastermix, and sterile plasticware were placed in their respective holders within OTTO’s work envelope. Through the companion software, OTTO was programmed to serial dilute cDNA from a control group, combine the mastermix with the primers, and mix all the reagents within the 96-well plate to form a 20-μL reaction volume (Fig. 5A). Pipette tips were automatically switched when necessary to prevent cross contamination. Further, OTTO pipetted up and down five times to mix the reagents prior to drawing the required volume into the pipette tip. The RSC generated from this experiment had an R^2^ value of 0.998, which exceeds the requirements for qPCR^8^ (Fig. 5B).

## Discussion

qPCR is a sensitive technique for detecting genetic material that has a broad range of applications, from testing for the presence of pathogens to analyzing expression of genes. However, this sensitivity means that small human errors when preparing samples for qPCR, such as the failure to thoroughly mix reagents or inconsistent pipetting technique, can result in significant variability. Furthermore, qPCR preparation is a time-consuming and tedious technique. Commercial automated liquid handlers that address these problems are available but expensive and based on proprietary designs. At the low end of the price range (> $ 5,000) is the OT-2 manufactured by Opentrons (https://opentrons.com/). While the OT-2 is marketed as an open-source liquid device, it is constructed from components that cannot be easily sourced and requires custom micropipettes. Sourcing the parts and building the device may be perceived as complicated by researchers who are unfamiliar with CNC technology. However, the rise of Maker Culture and digital fabrication has enabled our lab and others with limited backgrounds in CNC machines to design affordable and reliable alternatives to commercial instruments.

Recently, the syringe pump has become a popular DIY project for accurately dispensing fluid at a low cost^15–17^. One of the most inexpensive pump designs utilizes commercially available latex balloons to control the rate of fluid flow by varying the size and thickness of the balloon^28^. Reinforcing the balloon with elastane fibers enables operation at pressures sufficient to achieve shear stresses required for investigating the mechanobiology of human aortic endothelial cells^29^. While the affordability, simplicity, and scalability of balloon pumps renders them widely applicable to microfluidic devices, pressure loss from the balloon can reduce the flow rate over time^28^. Consequently, more expensive approaches, such as an Arduino microcontroller, are needed to achieve the constant aspiration volumes required for the micropipetting steps in preparation of samples for qPCR. Previous open-source microcontroller designs have deployed a stepper motor and lead screw actuator that depresses the plunger of a syringe to drive axial fluid flow at a fixed location. OTTO expands upon these relatively simple fixed axial flow devices. Multiple linear actuators controlled by an Arduino not only accurately dispense small volumes of liquid but also position them in 3D space. While the linear actuator in a syringe pump moves relatively slowly^15^, the speed of the actuators responsible for positioning OTTO’s micropipette directly affects the duration of sample preparation. This speed criteria dictated our choice of linear actuators as we attempted to balance reliability and speed by using a combination of belt- and lead screw-driven actuators.

Building a series of linear rails to form a platform capable of 3D motion is well known in the maker community^30^. However, a 3D motion liquid handler that accommodates potential sources of error associated with liquid handling, such as bent or improperly seated pipette tips, and embraces the open-source, modularity, and affordability principles of the Maker Movement has not been previously reported. Defects in the shape of pipette tips further complicated the design of error-free 3D motion. Our inclusion of the floating head design and tip alignment sensors dramatically improved the reliability of sample preparation with OTTO. If multiple tips fail to load or an obstruction prevents movements in the Z-axis, then OTTO will pause and inform the user, which allows pipette tips and labware that are not optimized for automation to be used. In future studies, the use of computer vision to detect loading and obstruction errors simultaneously would potentially eliminate the need for moving micropipettes and sensors. Low-cost solutions integrating smartphones and microfluidic devices^31,32^ would allow for automatic sensing of properly loaded pipette tips and also recording for easier troubleshooting. The addition of these low-cost computer vision approaches is anticipated to further enhance the reliability of OTTO.

Proof-of-concept experiments demonstrated that the volumetric accuracy of OTTO is comparable to that of the micropipette^27^ and that consistent qPCR standard curves can be generated at processing times comparable to manual operation^8^. OTTO is a low-wear system designed using durable components that are affordable and easy to replace. Because the parts are long-lasting, accelerated endurance testing is required to determine the long-term durability of OTTO. To assist other laboratories with building OTTO, we have created an online 3D CAD model that includes all the mechanical components used to construct OTTO (available at https://OpenLiquidHandler.com and in the online Supplementary Information 5). This 3D model is fully interactable, allowing prospective builders to explore and modify OTTO without purchasing a single component. The code and 3D-printable components are freely available in file formats that are readily modifiable, including the ability to sense the presence and alignment of transparent pipette tips. Future design improvements, such as the inclusion of computer vision, could be uploaded to the http://OpenLiquidHandler.com website as they become available. This accessibility is anticipated to promote adoption and improvement of OTTO by both the scientific and Maker communities.

## Conclusion

There are multiple benefits to automating sample preparation in wet laboratories, including increased throughput and reproducibility. To address this need, we have developed OTTO, the affordable liquid handler for automated micropipetting. Embracing the Maker Culture, this DIY design uses common open-source components and its assembly is fully documented online (https://OpenLiquidHandler.com). OTTO is not only a platform for automated liquid handling but also a platform to educate researchers about the benefits of CNC technology.

## Supplementary information

Supplementary Information 1.

Supplementary Information 2.

Supplementary Information 3.

Supplementary Information 4.

Supplementary Video.

Supplementary Information 5.

Supplementary Information 6.

## Data Availability

Testing data is available at https://OpenLiquidHandler.com/data.
