# Supplementary material for: Principles of computer-controlled linear motion applied to an open-source affordable liquid handler for automated micropipetting
Source: Sci Rep. 2020 Aug 12;10:13663. doi: 10.1038/s41598-020-70465-5 (PMC7424513; doi:10.1038/s41598-020-70465-5)
Supplement: Supplementary file 1 — Supplementary Information 1. [file 41598_2020_70465_MOESM1_ESM.docx]

**Principles of computer-controlled linear motion applied to an open-source affordable liquid handler for automated micropipetting**

David C. Florian^1,2^, Mateusz Odziomek^1^, Cerie L. Ock^1^, Hannah Chen^1^, & Scott A. Guelcher* ^1,2,3^

^1^Department of Biomedical Engineering, Vanderbilt University, Nashville, TN, 37235, USA

^2^Center for Bone Biology, Vanderbilt University Medical Center, Nashville, TN, 37232, USA

^3^Department of Chemical and Biomolecular Engineering, Vanderbilt University, Nashville, TN 37235, USA

* To whom correspondence should be addressed: Scott Guelcher ([scott.guelcher@Vanderbilt.edu](mailto:scott.guelcher@Vanderbilt.edu))

**Supplementary materials:**

The bill of materials, list of printable parts, mechanical assembly, stepper motor wiring schematic, description of through-beam sensors, wiring diagrams, description of the Tkinter GUI interface, and G-code are provided as Supplementary Tables and Figures.

The following files have been uploaded as Supplementary materials to assist other laboratories with building OTTO. These files are also available at OpenLiquidHandler.com.

| File name | Description |
| --- | --- |
| 3D-Printable-Components-STEP-Files.zip | Design files for 3D-printable components (STEP files) |
| 3D-Printable-Components-STL-Files.zip | Design files for 3D-printable components (STL files) |
| OTTO- The Open-Source Automatic Liquid Handler.mp4 | Video of OTTO in operation |
| OTTO-Assembly.zip | Assembly files |
| G-code-Interpreter-OTTO-Arduino-Firmware.ino | Arduino firmware |
| OTTO-Companion-Software.zip | Python program that runs OTTO |

**Supplementary Table S1**. Bill of materials for the mechanical and electrical components required to build OTTO. The prices listed in the table were compiled on June 1, 2020. The components were selected to minimize the number of vendors to source all the parts. More expensive components like the Balluff fork sensors and the Haydon-Kerk NEMA 11 linear actuator can be found at lower prices on the second-hand market. The pipette tip sensors can be constructed from either Balluff fork sensors for contactless probing or from mechanical switches for a more cost affordable solution (see **Figure S2**).

| Part Name | Part Number | Vendor | Quantity | Unit  Cost | Total Cost |
| --- | --- | --- | --- | --- | --- |
| MECHANICAL COMPONENTS | | | | | |
| 500mm C-Beam® Linear Actuator Bundle | 995-Bundle | [OpenBuilds](https://openbuildspartstore.com/c-beam-linear-actuator-bundle/) | 2 | $119.99 | $239.98 |
| 500mm C-Beam® Linear Rail | 10-LP | [OpenBuilds](https://openbuildspartstore.com/c-beam-linear-rail/) | 2 | $17.99 | $35.98 |
| 500mm V-Slot® 20x40 Linear Rail | 155-LP | [OpenBuilds](https://openbuildspartstore.com/v-slot-20x40-linear-rail/) | 2 | $6.99 | $13.98 |
| 500mm V-Slot® 20x60 Linear Rail | 290-LP | [OpenBuilds](https://openbuildspartstore.com/v-slot-20x60-linear-rail/) | 1 | $4.99 | $4.99 |
| 500mm V-Slot® 20x80 Linear Rail | 165-LP | [OpenBuilds](https://openbuildspartstore.com/v-slot-20x80-linear-rail/) | 4 | $12.99 | $51.96 |
| 250mm C-Beam® Linear Rail | 05-LP | [OpenBuilds](https://openbuildspartstore.com/c-beam-linear-rail/) | 2 | $8.99 | $17.98 |
| 250mm V-Slot® 20x20 Linear Rail | 280-LP | [OpenBuilds](https://openbuildspartstore.com/v-slot-20x20-linear-rail/) | 4 | $3.29 | $13.16 |
| V-Slot® Gantry Plate - Universal | 621 | [OpenBuilds](https://openbuildspartstore.com/v-slot-gantry-plate-universal/) | 2 | $11.99 | $23.98 |
| C-Beam® Gantry Plate - Double Wide | 2026 | [OpenBuilds](https://openbuildspartstore.com/c-beam-gantry-plate-double-wide/) | 1 | $13.99 | $13.99 |
| Motor Mount Plate - NEMA 17 Stepper Motor | 575 | [OpenBuilds](https://openbuildspartstore.com/motor-mount-plate-nema-17-stepper-motor/) | 1 | $6.99 | $6.99 |
| T Joining Plate | 615 | [OpenBuilds](https://openbuildspartstore.com/t-joining-plate/) | 12 | $3.99 | $47.88 |
| C-Beam® End Mount | 965 | [OpenBuilds](https://openbuildspartstore.com/c-beam-end-mount/) | 2 | $8.99 | $17.98 |
| GT2-2M Timing Pulley - 20 Tooth | 210 | [OpenBuilds](https://openbuildspartstore.com/gt2-2m-timing-pulley-20-tooth/) | 1 | $5.99 | $5.99 |
| 8mm Metric Acme Lead Screw | 25-LP | [OpenBuilds](https://openbuildspartstore.com/8mm-metric-acme-lead-screw/) | 1 | $10.99 | $10.99 |
| Anti-Backlash Nut Block | 1055-Set | [OpenBuilds](https://openbuildspartstore.com/anti-backlash-nut-block-for-8mm-metric-acme-lead-screw/) | 1 | $9.99 | $9.99 |
| GT2-2M Timing Belt - By the Foot | 470 | [OpenBuilds](https://openbuildspartstore.com/gt2-2m-timing-belt-by-the-foot/) | 5 | $2.49 | $12.45 |
| Black Angle Corner Connector | 540 | [OpenBuilds](https://openbuildspartstore.com/black-angle-corner-connector/) | 12 | $2.99 | $35.88 |
| 90 Degree Joining Plate | 610 | [OpenBuilds](https://openbuildspartstore.com/90-degree-joining-plate/) | 2 | $4.99 | $9.98 |
| Flanged Bearing 688ZZ 8x16x5 | 2215 | [OpenBuilds](https://openbuildspartstore.com/flanged-bearing-688zz-8x16x5/) | 2 | $1.49 | $2.98 |
| 40mm Aluminum Spacers (10 Pack) | 95-Pack | [OpenBuilds](https://openbuildspartstore.com/aluminum-spacers-10-pack/) | 1 | $7.89 | $7.89 |
| 6mm Aluminum Spacers (10 Pack) | 90-Pack | [OpenBuilds](https://openbuildspartstore.com/aluminum-spacers-10-pack/) | 1 | $3.39 | $3.39 |
| 6mm Eccentric Spacer | 226 | [OpenBuilds](https://openbuildspartstore.com/eccentric-spacer/) | 6 | $1.99 | $11.94 |
| 8mm Lock Collar | 840 | [OpenBuilds](https://openbuildspartstore.com/lock-collar/) | 2 | $1.19 | $2.38 |
| Xtreme Solid V Wheel Kit | 465 | [OpenBuilds](https://openbuildspartstore.com/xtreme-solid-v-wheel-kit/) | 4 | $6.99 | $27.96 |
| Delrin Mini V Wheel Kit | 495 | [OpenBuilds](https://openbuildspartstore.com/delrin-mini-v-wheel-kit/) | 8 | $4.49 | $35.92 |
| 500mm Drag Chain Cable Carrier | 2455 | [OpenBuilds](https://openbuildspartstore.com/drag-chain-cable-carrier/) | 3 | $7.99 | $23.97 |
| Rubber Feet Set (4 Pack) | 2645-Set | [OpenBuilds](https://openbuildspartstore.com/rubber-feet-set-4-pack/) | 1 | $7.99 | $7.99 |
| M5 x 8mm Bolt (10 Pack) | 946-pack | [OpenBuilds](https://openbuildspartstore.com/low-profile-screws-m5-10-pack/) | 5 | $0.99 | $4.95 |
| M5 x 40mm Bolt (10 Pack) | 135-pack | [OpenBuilds](https://openbuildspartstore.com/low-profile-screws-m5-10-pack/) | 1 | $1.69 | $1.69 |
| M5 Tee Nuts (10 Pack) | 536-Pack | [OpenBuilds](https://openbuildspartstore.com/tee-nuts-m5-10-pack/) | 5 | $2.99 | $14.95 |
| T Joint Plates (10 Pack) | N/A | [Amazon](https://www.amazon.com/dp/B07CXNMLDQ/ref=cm_sw_em_r_mt_dp_U_clm.EbB7E7FWW) | 2 | $12.99 | $25.98 |
| SK8 Linear Rod Clamps (4 Pack) | N/A | [Amazon](https://www.amazon.com/dp/B07QS7Z8MK/ref=cm_sw_em_r_mt_dp_U_nF78EbHSYCBK5) | 1 | $11.29 | $11.29 |
| 8mm Linear Rods x 150mm (2 pack) | N/A | [Amazon](https://www.amazon.com/dp/B07SYYDGFM/ref=cm_sw_em_r_mt_dp_U_.wpOEbPGTMW74) | 1 | $8.30 | $8.30 |
| SCS8LUU Linear Ball Bearing Slide | N/A | [Amazon](https://www.amazon.com/dp/B07X7Z96TC/ref=cm_sw_em_r_mt_dp_U_eYk.Eb44DSNPY) | 2 | $11.09 | $22.18 |
| Compression Springs | 94125K219 | [McMaster-Carr](https://www.mcmaster.com/94125k219) | 2 | $3.47 | $6.94 |
|  |  |  |  | **Total:** | **$794.83** |
| ELECTRICAL COMPONENTS | | | | | |
| NEMA 17 Stepper Motor | 623 | [OpenBuilds](https://openbuildspartstore.com/nema-17-stepper-motor/) | 1 | $17.99 | $17.99 |
| NEMA 23 Stepper Motor | 518 | [OpenBuilds](https://openbuildspartstore.com/nema-23-stepper-motor/) | 3 | $27.99 | $83.97 |
| Limit Switch Kit | 2805-Kit | [OpenBuilds](https://openbuildspartstore.com/xtension-limit-switch-kit/) | 4 | $6.29 | $25.16 |
| NEMA 11 Linear Actuator | 28H41-2.1-907 | [Haydon Kerk](https://prototypes.haydonkerk.com/ecatalog/hybrid-linear-actuators/en/linear-actuator-28H41-2.1-907) | 1 | $192.97 | $192.97 |
| Arduino Due | 1050-1049-ND | [Digi-key](https://www.digikey.com/product-detail/en/arduino/A000062/1050-1049-ND/3712582) | 1 | $37.40 | $37.40 |
| TMC2660-BOB Stepper Drivers | 1460-1245-ND | [Digi-key](https://www.digikey.com/products/en?keywords=tmc2660-bob) | 5 | $19.95 | $99.75 |
| Mean Well 24V 14.6A Regulated DC Power Supply | LRS-350-24 | [Amazon](https://www.amazon.com/dp/B07VRK86SP/ref=cm_sw_em_r_mt_dp_U_nppOEb3SJP9R0) | 1 | $29.95 | $29.95 |
| 24V to 3.3V Optocoupler | DST-1R8P-P | [Amazon](https://www.amazon.com/dp/B06XKYS8VC/ref=cm_sw_em_r_mt_dp_U_HupOEbRYJPJQ8) | 1 | $10.49 | $10.49 |
| Breadboard (6 Pack) | N/A | [Amazon](https://www.amazon.com/dp/B07LFD4LT6/ref=cm_sw_em_r_mt_dp_U_jJQ-EbQHVWPKY) | 1 | $8.99 | $8.99 |
| Busbar (6 Pack) | N/A | [Amazon](https://www.amazon.com/dp/B07CLR3MLK/ref=cm_sw_em_r_mt_dp_U_fMQ-Eb1NES7H0) | 1 | $12.49 | $12.49 |
| AC Power Socket w/ Fuse | N/A | [Amazon](https://www.amazon.com/dp/B01FREPYRM/ref=cm_sw_em_r_mt_dp_U_MQQ-EbXM6JSX5) | 1 | $6.99 | $6.99 |
| Emergency Stop Button (22mm panel mount) | N/A | [Amazon](https://www.amazon.com/dp/B019DSZWPC/ref=cm_sw_em_r_mt_dp_U_eeQ-EbNGWWRK0) | 1 | $2.84 | $2.84 |
|  |  |  |  | **Total:** | **$528.99** |
| PIPETTE TIP SENSORS | | | | | |
| SS-5GL Mechanical Switch (8 Pack) | N/A | [Amazon](https://www.amazon.com/dp/B07XF9T2L2/ref=cm_sw_em_r_mt_dp_U_y1a.EbWKVCDWW) | 1 | $11.88 | $11.88 |
| Or | | | | | |
| Balluff Through-beam Fork Sensor | BGL 80A-009-S49 | [Balluff](https://www.balluff.com/local/us/productfinder/product/?key=BGL002T#/) | 2 | $342.28 | $684.56 |
|  |  |  |  |  |  |
| Total w/ Mechanical Switches: | **$1,335.7** |  |  |  |  |
|  |  |  |  |  |  |
| Total w/ Balluff Fork Sensors: | **$2,008.38** |  |  |  |  |

**Supplementary Table S2**. Printable parts required for the assembly of OTTO. A fused filament fabrication (FFF) 3D printer with a build volume of at least 203 x 232 x 203 mm is required to print all the parts. Approximately 3 kg of filament is needed to print the required number of parts at 20% infill. Polylactic acid (PLA) is a suitable print material. However, it is recommended that the Floating Head Plate is machined out of aluminum or printed out of a stiff filament (e.g., a carbon fiber composite) for increased rigidity of the floating head assembly. STL and STEP files for each component is provided as separate supplemental material.

| Part Name | Quantity | Function | Model |
| --- | --- | --- | --- |
| Pipette Clamp | 1 | The clamp holds the pipette within the housing. The clamp STEP file can be modified to hold different brands of micropipettes. | 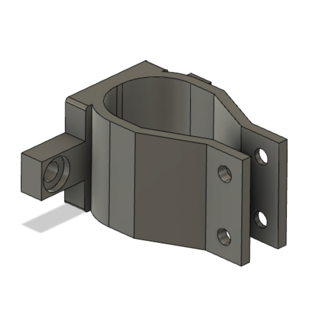 |
| Pipette Housing Body | 1 | The pipette housing contains the pipette clamp and NEMA 11 linear actuator, which actuates the pipette plunger. | 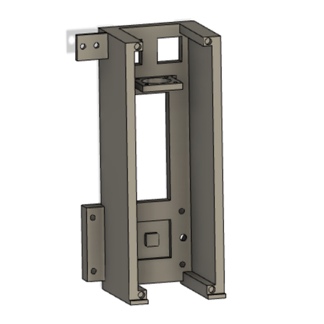 |
| Pipette Housing Top Plate | 1 | The top plate prevents debris from getting into the pipette housing and retains the face plate. | 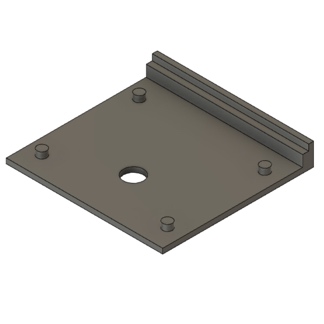 |
| Pipette Housing Face Plate | 1 | The face plate prevents debris from getting into the pipette housing. | 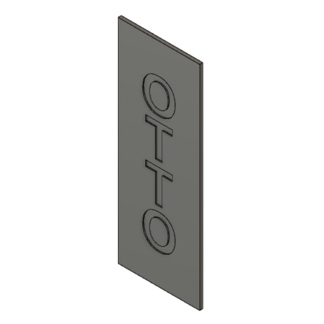 |
| Floating Head Plate | 1 | The rod clamps are fastened to the floating head plate, which is attached to the Z-axis. This plate can be machined out of aluminum or 3D printed with a stiff filament, such as a carbon fiber composite. | ­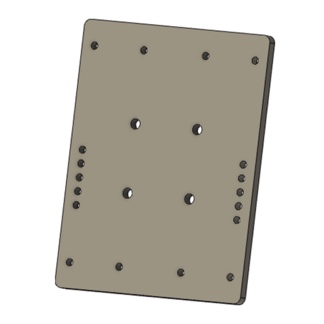 |
| Chiller Block Holder | 1 | The chiller block holder is located on the work table. It holds an aluminum cooler or ice block, which keeps well plates cold. | 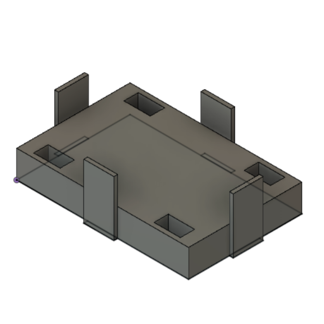 |
| 96 Well Plate Holder  (OPTIONAL) | 1 | The 96 well plate holder can be used in place of the chiller block holder if OTTO is in a cold room or if sample evaporation is not a concern. | 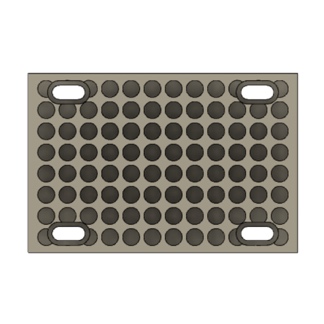 |
| Epi Tube Holder | 1 | The epi tube holder is located on the work table. It holds microcentrifuge tubes of primers and strips of PCR tubes containing cDNA. | 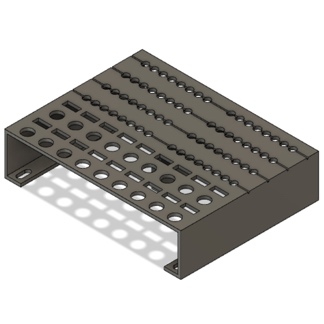 |
| Pipette Tip Holder | 2 | The pipette tip holder is located on the work table. This holder can contain two boxes of pipette tips, and it can be modified for use with different sizes and brands of pipette tips. | 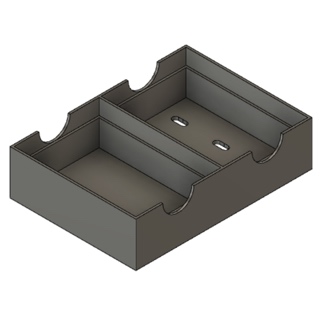 |
| Balluff Through-Beam Sensors Holder  (OPTIONAL) | 1 | The Balluff through-beam sensor holder maintains the positioning of two BGL 80A-009-S49 sensors on the work table. | 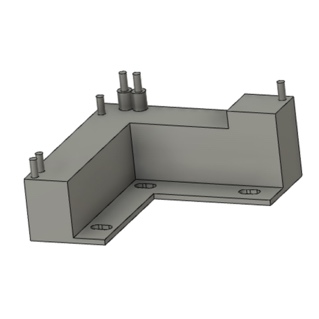 |
| Mechanical Switch Sensors Holder (OPTIONAL) | 1 | In place of the Balluff through-beam sensors holder, this holder can be used to probe the presence and concentricity of the pipette tips when SS-5GL mechanical switches are installed. | 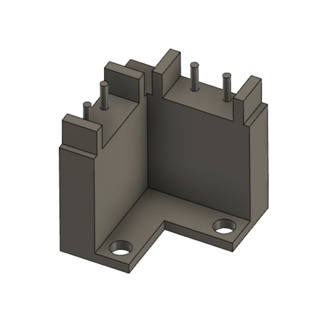 |
| Pipette Tip Remover | 1 | The pipette tip remover slides onto the pipette shaft between the pipette tip and the ejector, allowing OTTO to remove the tip when it moves up in the Z-direction. | 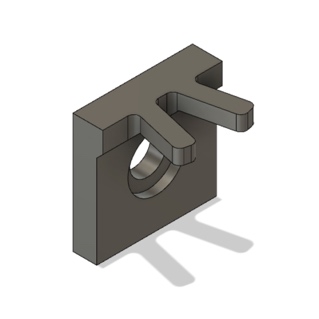 |
| Pipette Bin | 2 | The pipette bins collects the ejected tips. | 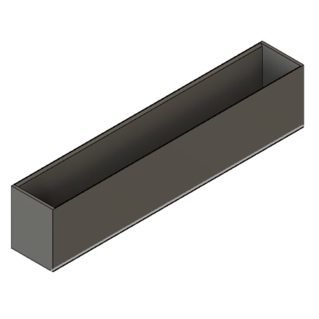 |
| Palette | 1 | The palette holds two common reagents (e.g., PCR Master Mix and water) in microcentrifuge tubes. The palette is attached to OTTO’s X-axis allowing the pipette quick access to frequently used solutions. | 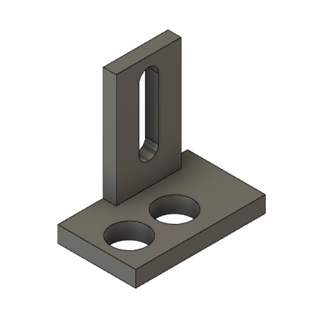 |
| Electronics Tray 1 | 1 | Electronics tray 1 slides into the aluminum extrusion channel beneath the workspace. This tray has mounting points for an Arduino Due and 4 TMC2660-BOB boards. | 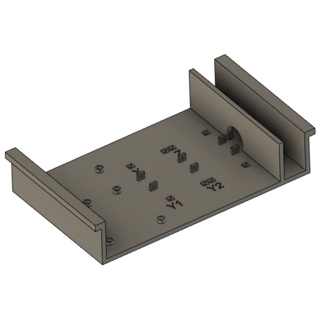 |
| Electronics Tray 2 | 1 | Electronics tray 2 slides into the aluminum extrusion channel beneath the workspace. This tray has mounting points for 2 TMC2660-BOB boards and 2 breadboards. | 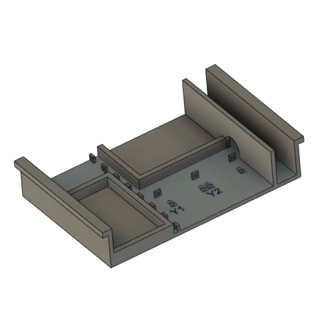 |
| Electronics Tray 3 | 1 | Electronics tray 3 slides into the aluminum extrusion channel beneath the workspace. This tray has mounting points for 2 24V to 3.3V optocouplers. | 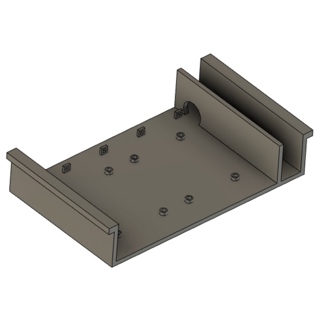 |
| Electronics Tray 4 | 1 | Electronics tray 4 slides into the aluminum extrusion channel beneath the workspace. This tray has mounting points for 1 breadboard and 2 busbars. | 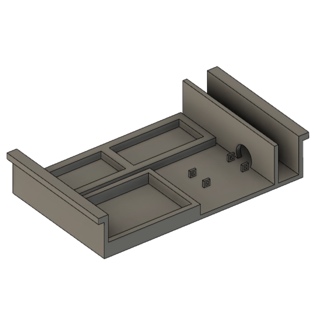 |
| Power Supply Mounting Slide | 2 | This mounting part attaches to either side of a Meanwell power supply, allowing the power supply to be suspended between two aluminum extrusions. | 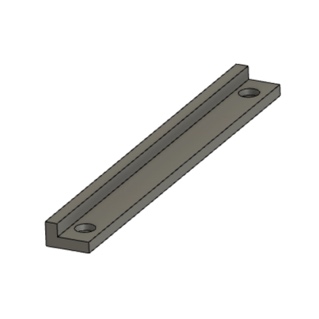 |
| Female AC Connector and Emergency Stop Button Housing | 1 | This housing contains the AC power socket with fuse and the emergency stop button. | 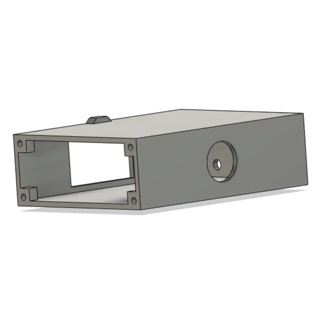 |
| Female AC Connector and Emergency Stop Play, and Pause Buttons Housing  (OPTIONAL) | 1 | This version of the housing contains cutouts for optional play and pause buttons in addition to the emergency stop button. The functionality of these buttons are not yet supported. | 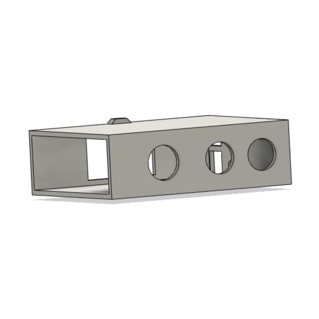 |
| Backplate | 1 | The backplate covers the open end of the female AC connector and emergency stop button housing. | 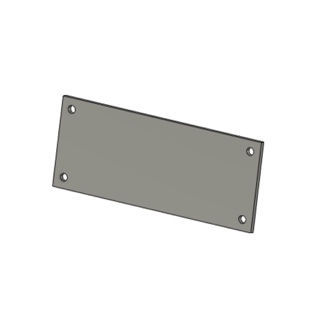 |
| Drag Chain Connector 1 | 3 | Drag chain connector 1 is used to anchor both ends of the X-axis drag chain and one end of the Z axis drag chain. | 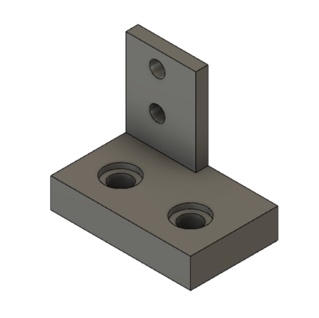 |
| Drag Chain  Connector 2 | 1 | Drag chain connector 2 is used to anchor one end of the Y-axis drag chain. | 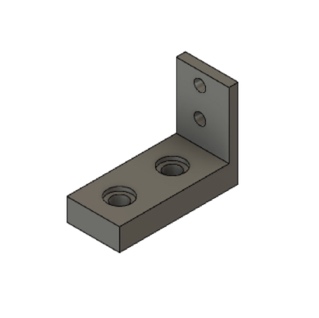 |
| Drag Chain  Connector 3 | 1 | Drag chain connector 3 is used to anchor the other end of the Y-axis drag chain, which is closest to the stepper motor. | 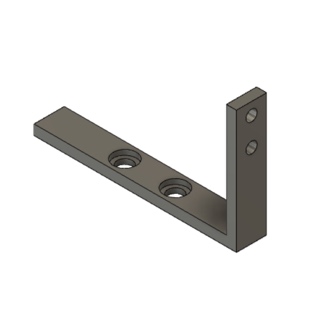 |


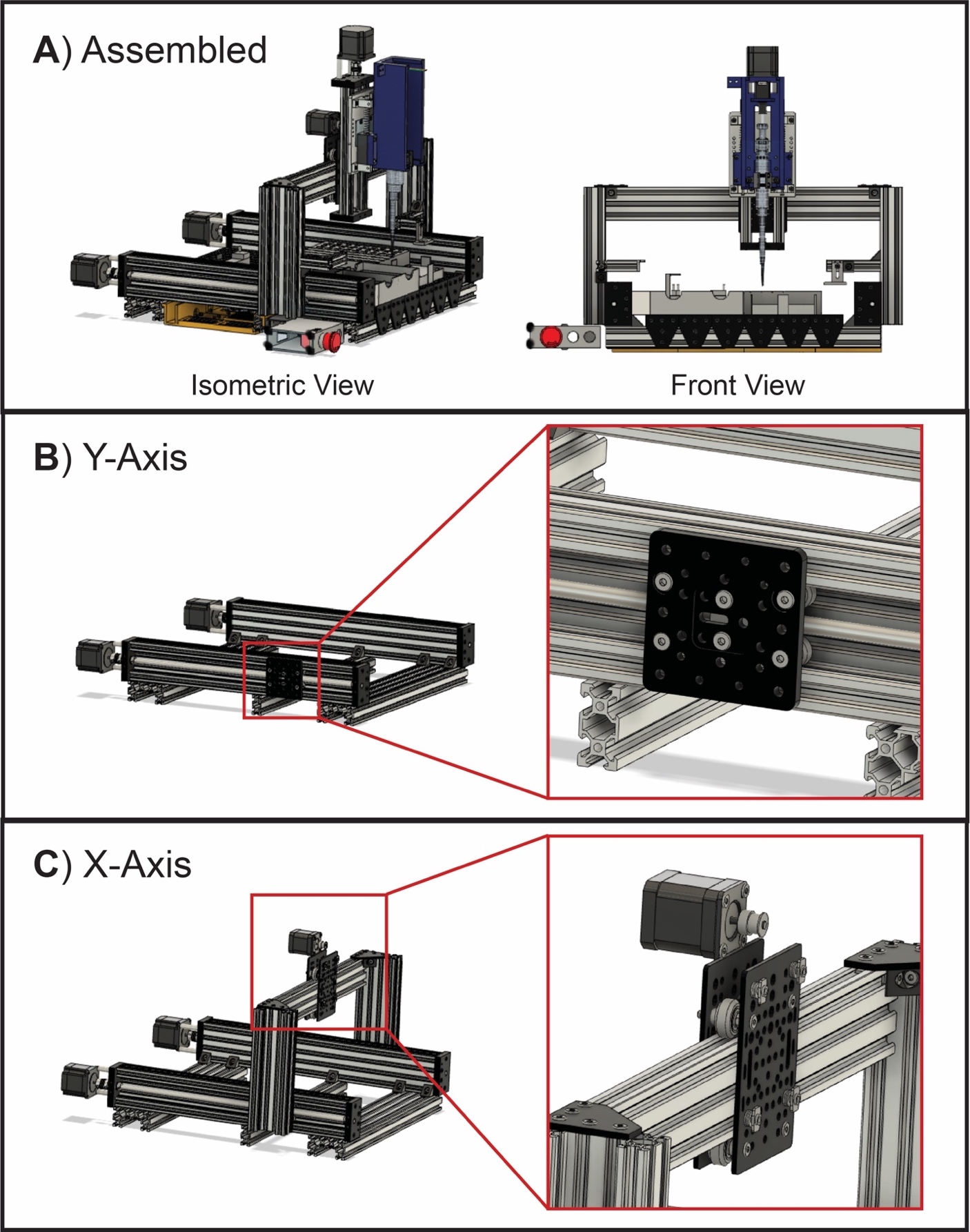


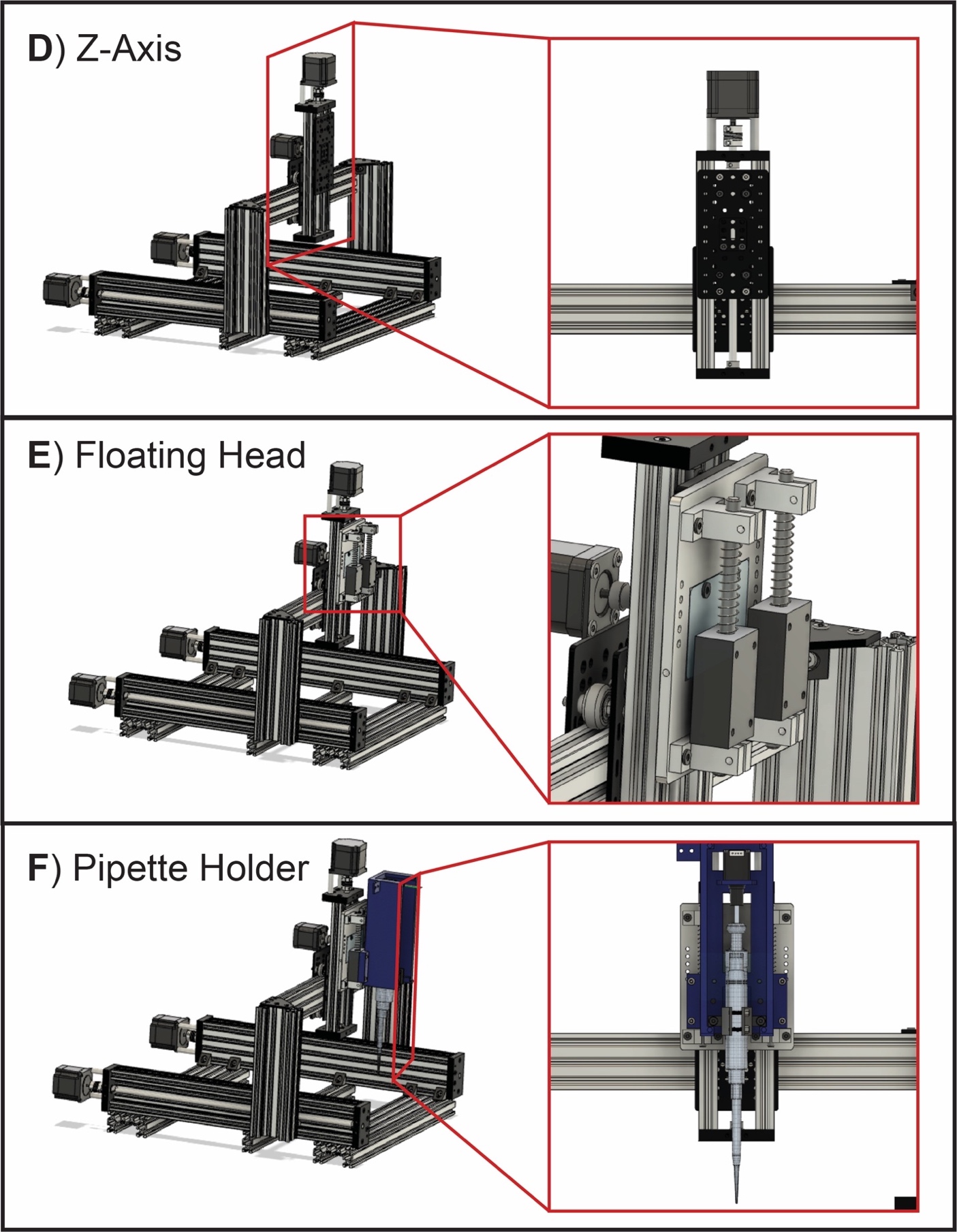


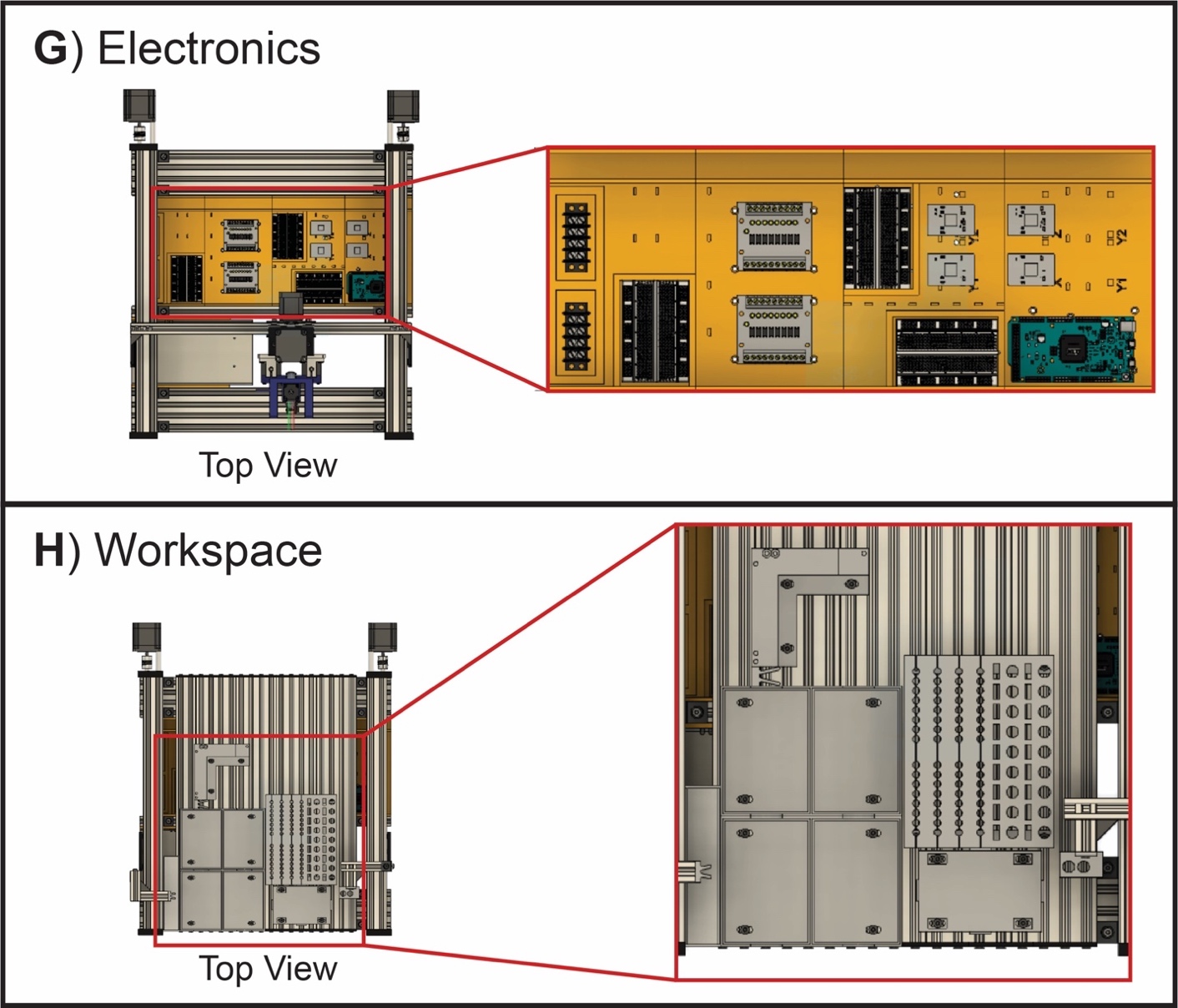


**Supplementary Figure S1.** Mechanical assembly of OTTO. An assembly (.f3d) file for OTTO is provided as separate supplemental material, which can be imported into Fusion 360 to generate a complete 3D model of OTTO. Referencing this model is the best approach for building OTTO. (A) Isometric and front views of a fully assembled OTTO. (B) The Y-axis consists of two lead screw linear actuators. These actuators are purchased as a DIY kit and detailed instructions for their assembly can be found on the [OpenBuilds website](https://openbuilds.com/builds/c-beam%E2%84%A2-linear-actuator.1955/). The Y-axis linear actuators are bolted down to the opposite ends of 2 C-Beams and a 20x40 aluminum extrusion to form the platform. (C) The X-axis is a belt and pinion linear actuator that is suspended above the workspace through C-Beams that are connected to the Y-axis carriages. More information about the assembly of belt and pinion linear actuators can be found on the [OpenBuilds website](https://openbuilds.com/builds/nema-17-belt-and-pinion-actuator.7499/). (D) The Z-axis is also a lead screw linear actuator, but it uses 20x20 linear rails instead of C-Beam to obtain a lower profile. (E) The floating head assembly is constructed from the floating head plate, SK8 linear rod clamps, 8mm linear rods, SCS8LUU linear ball bearing slides, and compression springs. (F) The micropipette is held within the pipette housing body by the pipette clamp. The pipette housing body is bolted to the SCS8LUU linear ball bearing slides. (G) The microcontroller, stepper drivers, optocouplers, busbars, and breadboards sit in the 4 electronics trays that slide in-between the aluminum extrusion of the platform. The 24V regulated DC power supply also mounts between the platform’s aluminum extrusion. (H) The workspace is constructed from 4 20x80 aluminum extrusions and 1 20x40 aluminum extrusion, which are laid across the platform and bolted down with T joint plates.


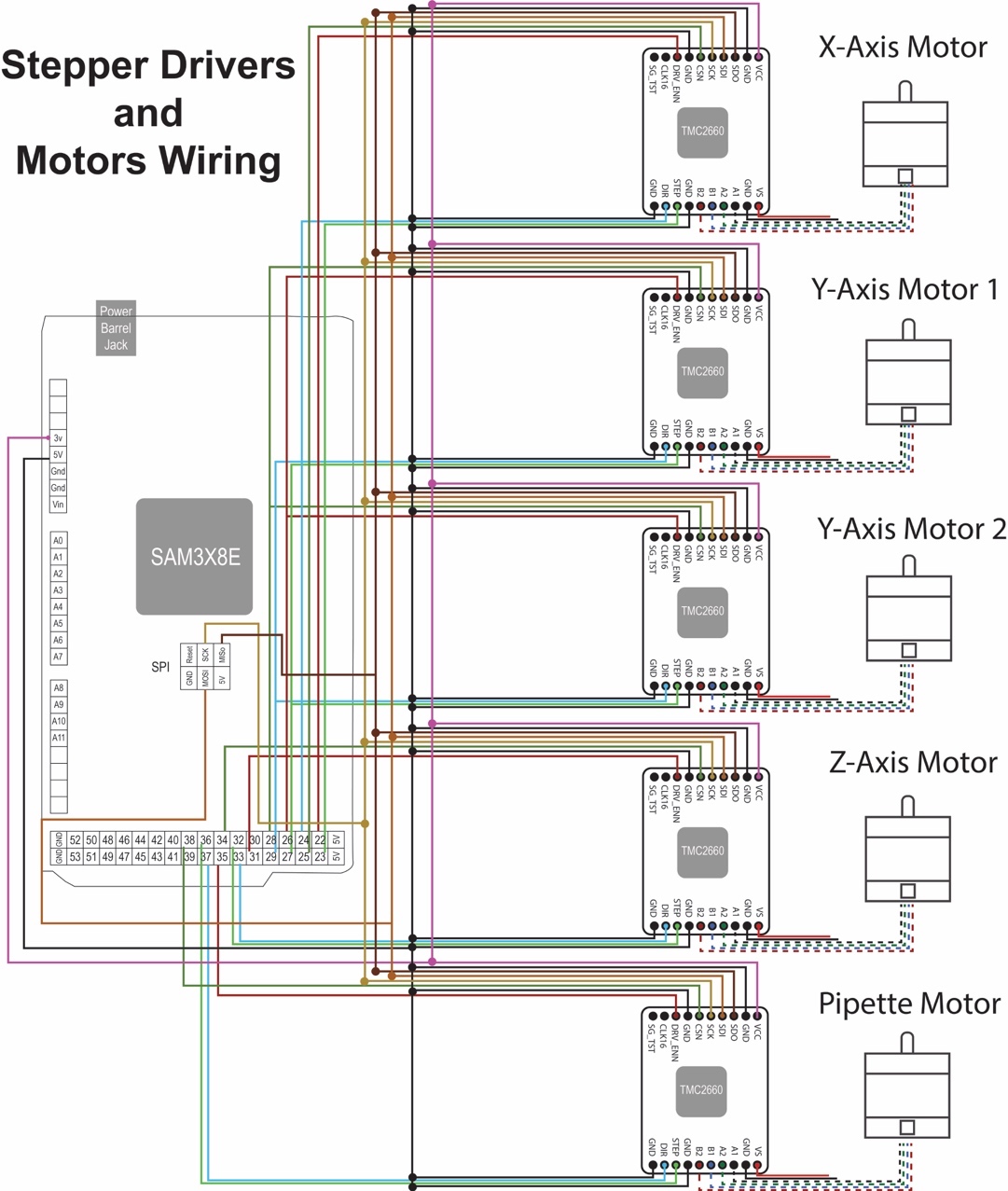


**Supplementary Figure S2.** Stepper motor wiring schematic. TMC2660 stepper drivers are used to coordinate the movement of the five stepper motors. These drivers communicate with the Arduino Due through a Serial Peripheral Interface (SPI) connection, which allows the motor current and microstepping levels to be set digitally. The TMC2660 are able to move the stepper motors at near silfent operation. Because of current requirements, the two Y axis motors are wired to their own stepper drivers. For synchronous movement between the two Y axis motors, the two TMC2660s share the same step, direction, enable, and chip select pins on the Arduino Due.

**
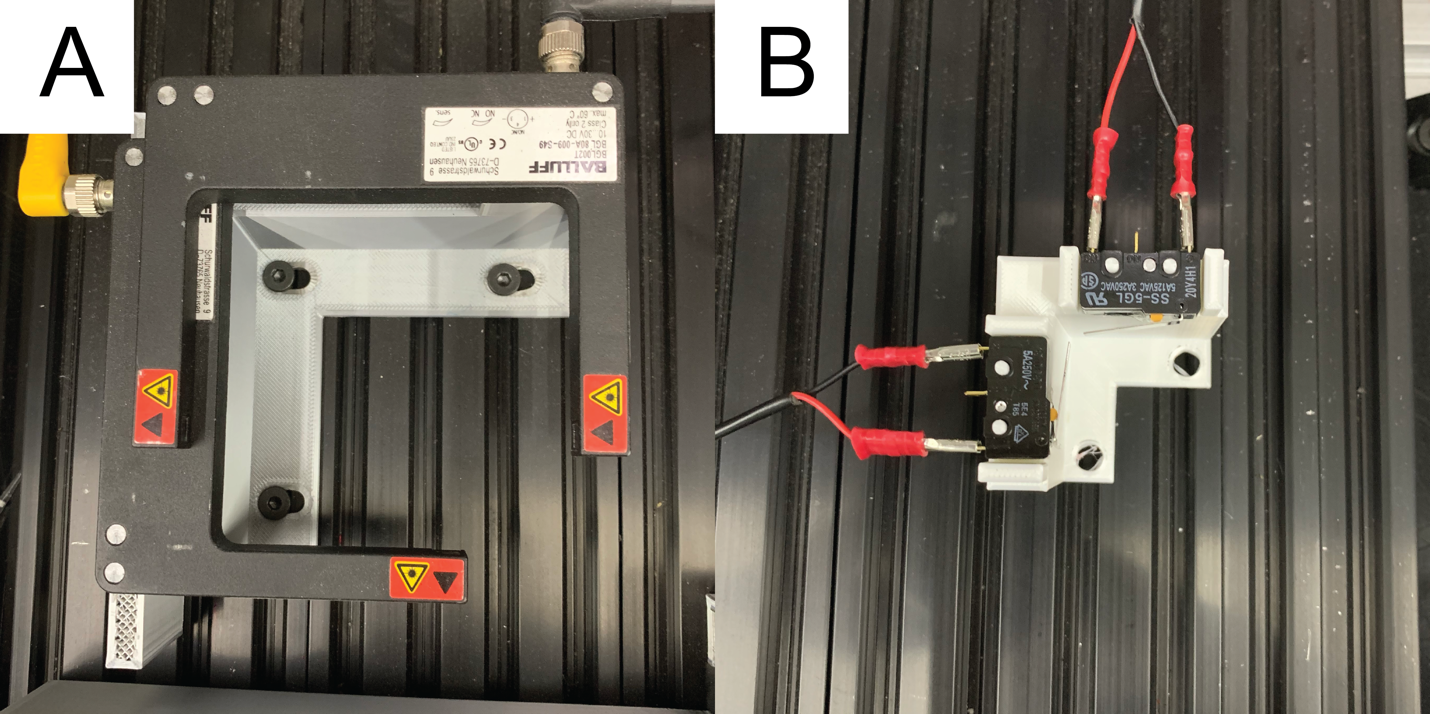
**

**Supplementary Figure S3.** Through-beam versus mechanical tip sensors. Sensing the presence and concentricity of the pipette tip is an optional liquid handling step that improves the reliability of OTTO. (A) Through-beam Balluff sensors are able to sense when a transparent object (e.g. pipette tip) passes through their beams by sensing changes in polarized light. This is a contactless process. However, the BGL 80A-009-S49 are expensive when not purchased from the second hand market. (B) Mechanical switches can check the presence and concentricity of the pipette tip at a much lower cost, but the tip must touch the switch’s lever arm, which could introduce contaminates to the tip.


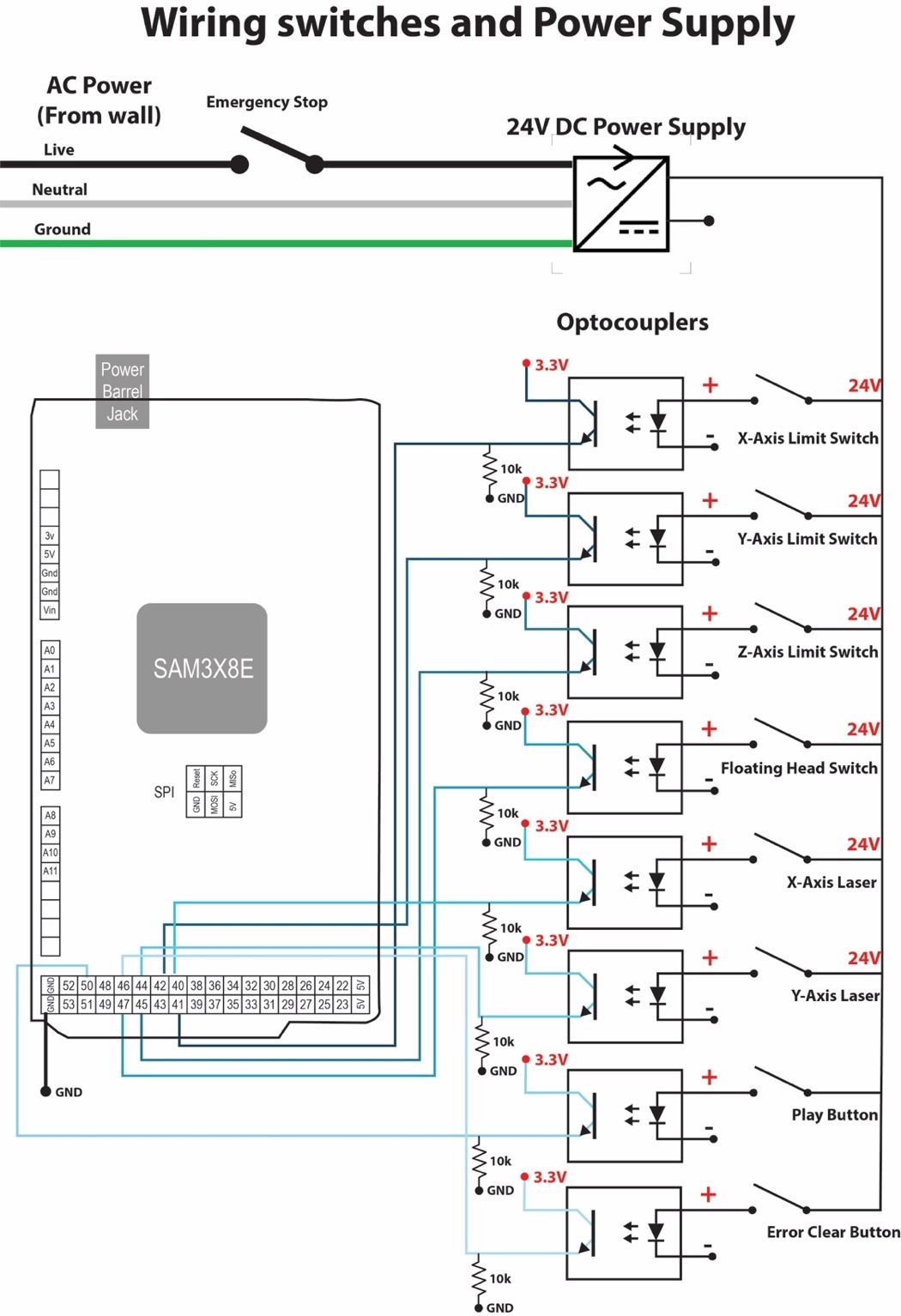


**Supplementary Figure S4.** Switch and sensor wiring. To minimize the effects of electrical noise, the switches, sensors, and buttons are supplied 24V. However, the Arduino Due can only tolerate 3.3V of input on its pins, so the 24V signal from the switches or sensors is converted to 3.3V through optocouplers. The 3.3V signal from the optocoupler is connected to the appropriate pin but also to ground through a 10k Ohm pull-down resistor to ensure a well-defined ground voltage. The pull-down resistors were wired on a breadboard to circumvent the need for soldering.

**Supplementary Table S3**. Wiring table. The Arduino Due runs a custom firmware known as G-Code Interpreter. This firmware allows the Arduino Due to coordinate the movement of the stepper motors and sense changes in the state of limit switches and sensors. For the Arduino Due to function properly the wiring of the stepper drivers, sensors, and switches to its pins need to match the those defined in the firmware. Therefore, the user can either wire their components according to this table or adjust the definitions within the firmware file found in the additional supplementary material.

| **STEPPER DRIVERS** | | | | | | | |
| --- | --- | --- | --- | --- | --- | --- | --- |
| **Stepper Driver Pins** | **Arduino Pins or Power Supply** | | | | | | |
|  | *X-Axis* | *Y-axis* | | *Z-Axis* | | | *P-Axis* |
| *VS* | 24V | 24V | | 24V | | | 24V |
| *GND* | GND | GND | | GND | | | GND |
| *VCC* | 3.3V | 3.3V | | 3.3V | | | 3.3V |
| *SDO* | MISO Pin | MISO Pin | | MISO Pin | | | MISO Pin |
| *SDI* | MOSI Pin | MOSI Pin | | MOSI Pin | | | MOSI Pin |
| *SCK* | SCK Pin | SCK Pin | | SCK Pin | | | SCK Pin |
| *Enable* | Digital Pin 22 | Digital Pin 26 | | Digital Pin 31 | | | Digital Pin 35 |
| *Step Pulse* | Digital Pin 23 | Digital Pin 27 | | Digital Pin 32 | | | Digital Pin 36 |
| *Direction Pulse* | Digital Pin 24 | Digital Pin 29 | | Digital Pin 33 | | | Digital Pin 37 |
| *Chip Select* | Digital Pin 25 | Digital Pin 28 | | Digital Pin 34 | | | Digital Pin 26 |
| **LIMIT SWITCHES** | | | | | | | |
| **Limit Switch Pins** | **Arduino Pins or Power Supply** | | | | | | |
|  | *X-Axis* | | *Y-axis* | | | *Z-axis* | |
| *Common* | 24V | | 24V | | | 24V | |
| *Normally Closed* | Digital Pin 41 | | Digital Pin 42 | | | Digital Pin 45 | |
| **FLOATING HEAD SWITCH** | | | | | | | |
| **Floating Head Pins** | **Arduino Pins or Power Supply** | | | | | | |
| *Common* | 24V | | | | | | |
| *Normally Closed* | Digital Pin 47 | | | | | | |
| **THROUGH-BEAM TIP SENSORS (OPTIONAL)** | | | | | | | |
| **Laser Sensors Pins** | **Arduino Pins or Power Supply** | | | | | | |
|  | *X-Axis Laser* | | | | *Y-Axis Laser* | | |
| *GND* | GND | | | | GND | | |
| *VCC* | 24V | | | | 24V | | |
| *Signal* | Digital Pin 44 | | | | Digital Pin 40 | | |
| **MECHANICAL TIP SENSORS (OPTIONAL)** | | | | | | | |
| **Mechanical Sensors Pins** | **Arduino Pins or Power Supply** | | | | | | |
|  | *X-Axis Mechanical Switch* | | | | *Y-Axis Mechanical Switch* | | |
| *Common* | 24V | | | | 24V | | |
| *Normally Closed* | Digital Pin 44 | | | | Digital Pin 40 | | |


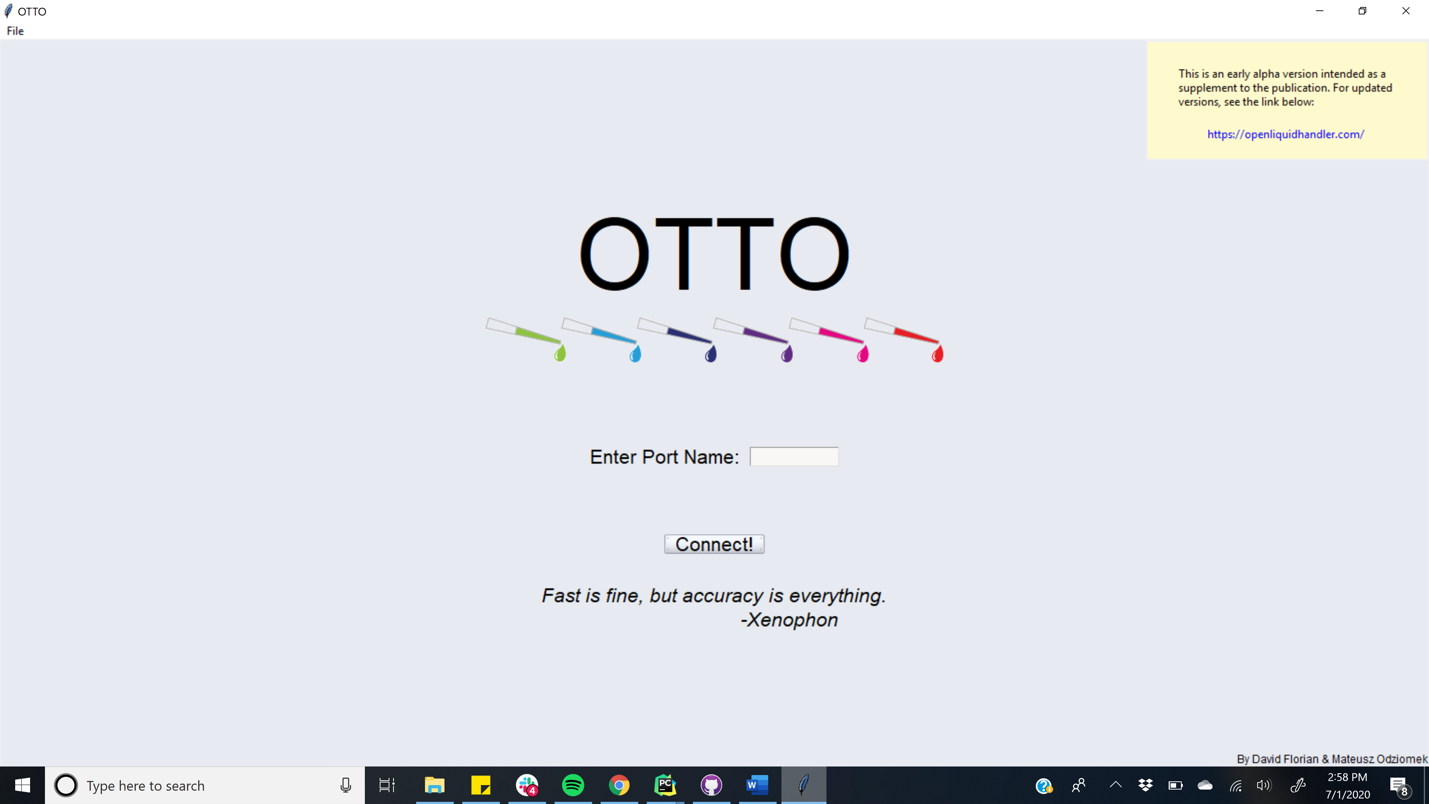


**Supplemental Figure S5.** Computer software connection screen. The graphical user interface for controlling OTTO is written in Python 3. The Python code is included in the supplemental material. Prior to running the code, the dependencies (e.g., tkinter, serial, etc.) will need to be installed. An executable file with the latest version of the software is available online ([www.openliquidhandler.com/software/](http://www.openliquidhandler.com/software/)), which simplifies the installation process. The GUI is currently optimized for a windows computer with a 1080p monitor. The connection screen asks for the serial communication port that the Arduino Due is connected to (e.g., COM4). Successful communication between the software program and the Arduino Due will allow the user to proceed to the next screen.


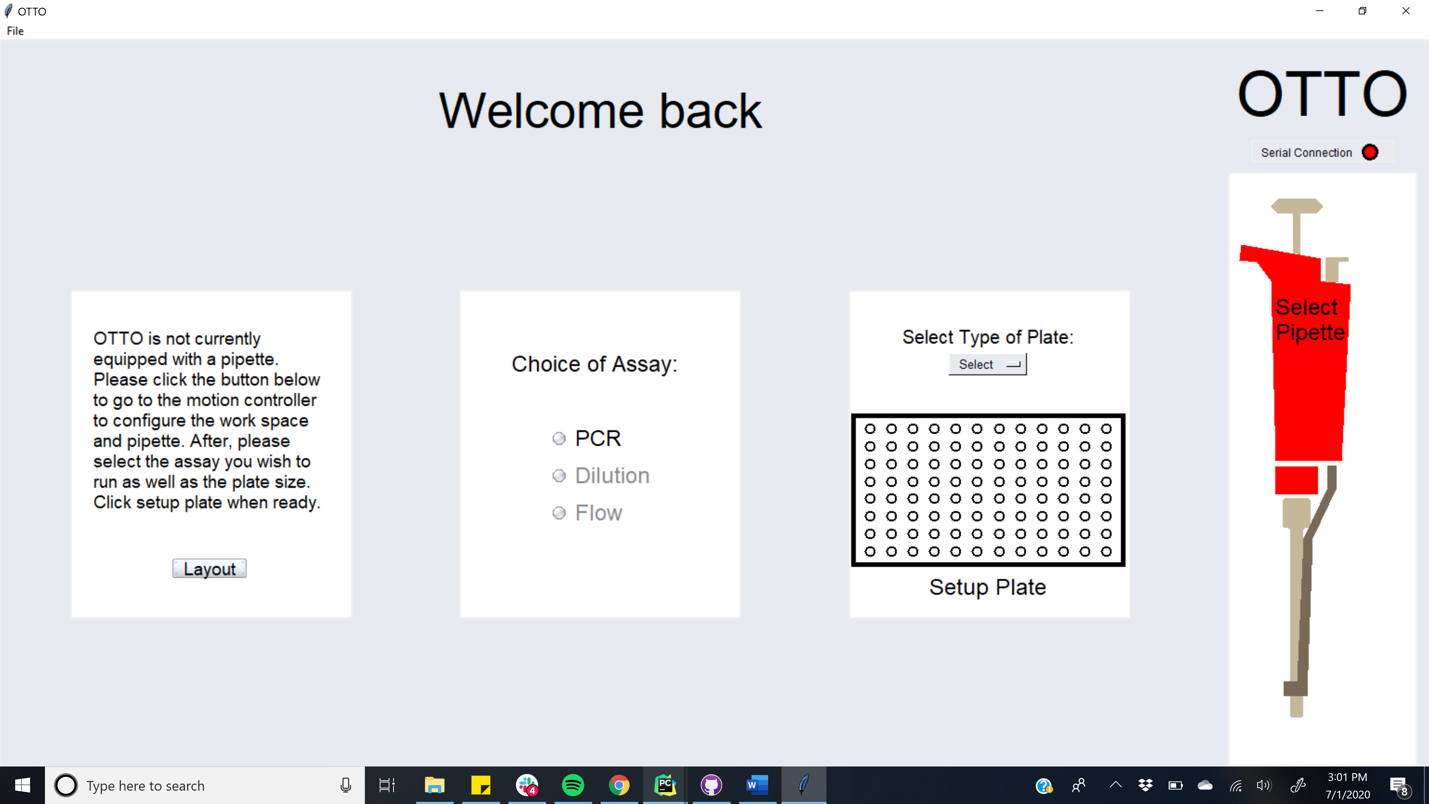


**Supplemental Figure S6.** Assay and plate selection page. After the software connects with the Arduino Due, the user is brought to the assay and plate selection page. The first time that the software is run the user will have to click the “Layout” button to calibrate OTTO. After calibrating OTTO (see **Figure S7**) subsequent runs of the software will not require the user to click the “Layout” button. The assay plate and selection page allows the user to select an assay and the type of plate or tubes to combine the reagents into. Currently, the stable version of the code only allows for PCR preparation to be performed in a 96 well plate. For more features, the reader is directed to [www.openliquidhandler.com/software/](http://www.openliquidhandler.com/software/) to install the most recent version of the software.


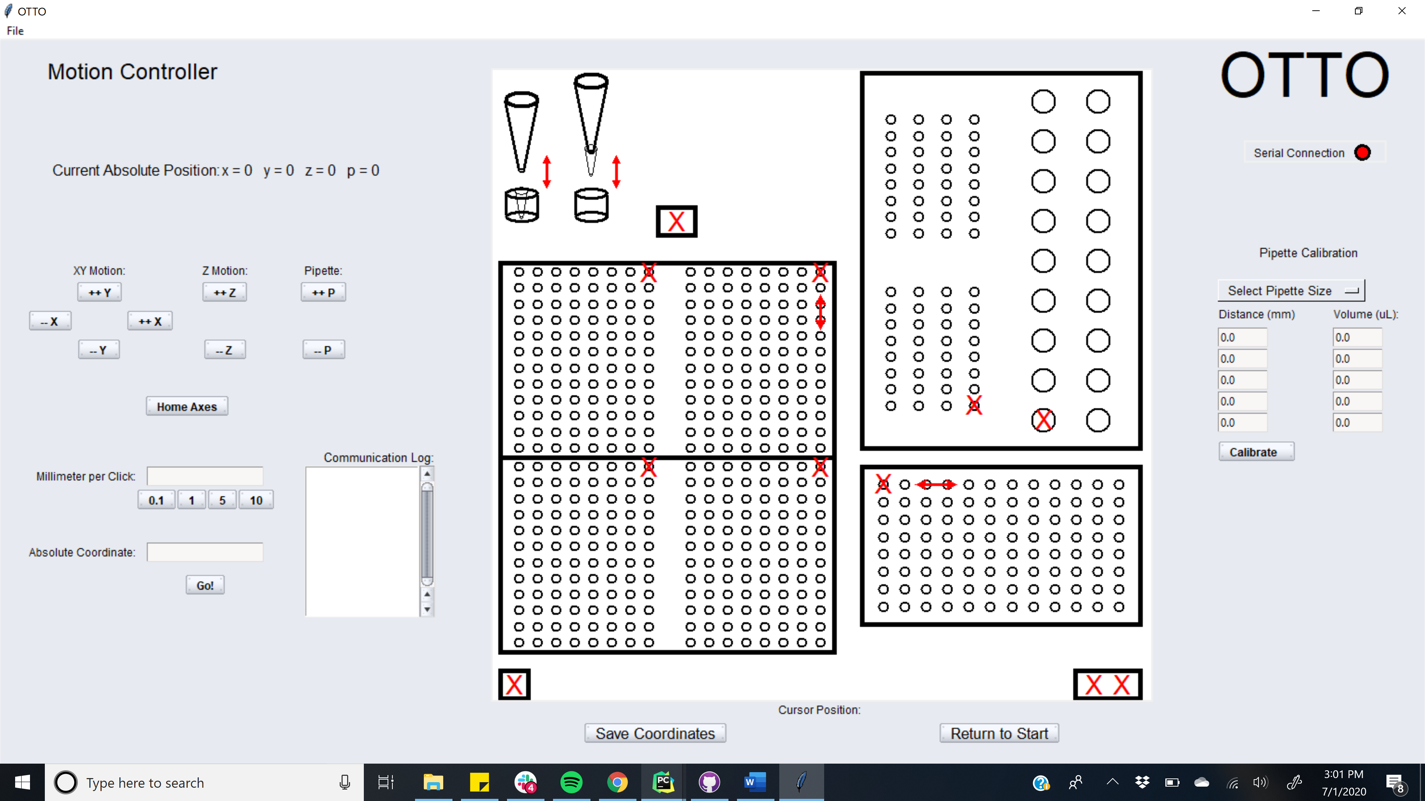


**Supplemental Figure S7.** The Motion Controller page is responsible for setting the location of the components within the work table and for calibrating the pipette. The user can move OTTO through relative coordinates by clicking on the buttons corresponding to the desired axis after selecting a value underneath the “Millimeter per Click” field. OTTO can also be moved by entering absolute coordinates into the “Absolute Coordinate” field, where each coordinate must be delineated by a space (e.g., X Y Z P). Red Xs indicate positional coordinates that are required for OTTO to locate pipette tips, reagents, sensors and other components for qPCR reactions. Arrows indicate spacing between components. Selecting a red X or arrow will open a dialogue providing further information and fields to input the absolute position of the marker.  After entering all the coordinates for each red X and arrow the user must press the “Save Coordinates” button at the bottom of the screen. The Motion Controller page is also used to calibrate the linear actuator that will engage the pipette plunger. The user must move the linear actuator through relative and/or absolute P coordinates and record the volume dispensed for a range of movements in the designated fields for accurate pipetting.


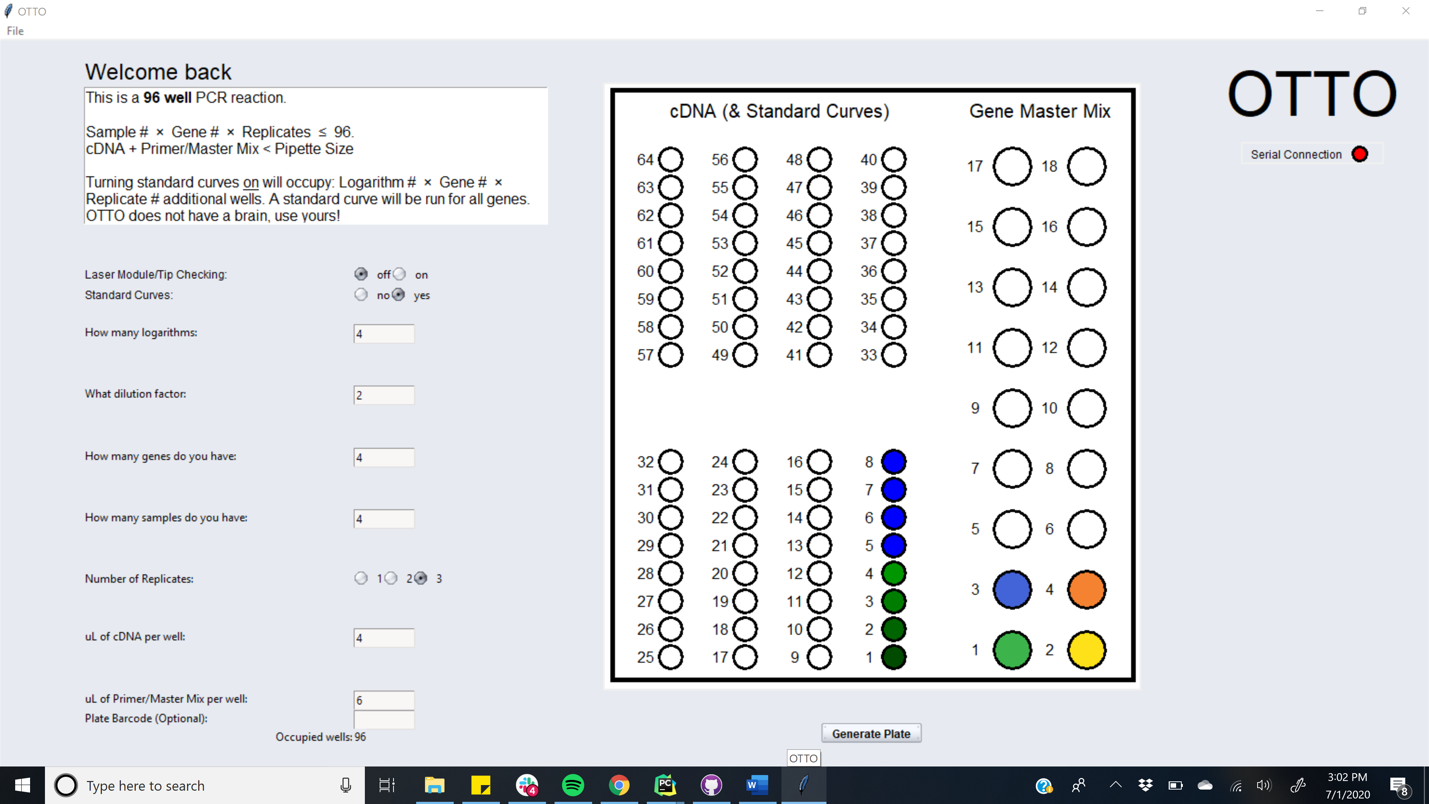


**Supplementary Figure S8.** The Plate Setup page is used to enter the parameters for the PCR reaction. Users have the option of turning on tip checking to increase reliability or turning it off to decrease run time. Users can also select whether to have OTTO generate a standard curve at a custom dilution factor and range. The number of genes, cDNA samples, and replicates also must be entered. This information is used to automatically generate a reagent map, which shows the user where reagents must be placed in the microcentrifuge tube holder. Both primers for each gene must be preloaded into the same microcentrifuge tube with an appropriate concentration of Master Mix. In future iterations of the software, OTTO will automatically add the appropriate volume of Master Mix. The plate setup page is designed for 96 well plates, and the “Occupied wells” counter at the bottom of the screen will turn red if the user inputs too many genes, samples, or replicates. The user must input the volume of cDNA and Primer/Master Mix to add to each well. If the volume is greater than the calibrated pipette can hold, then the software will throw an error. Clicking “Generate Plate” will create the G-code required for the PCR preparation.


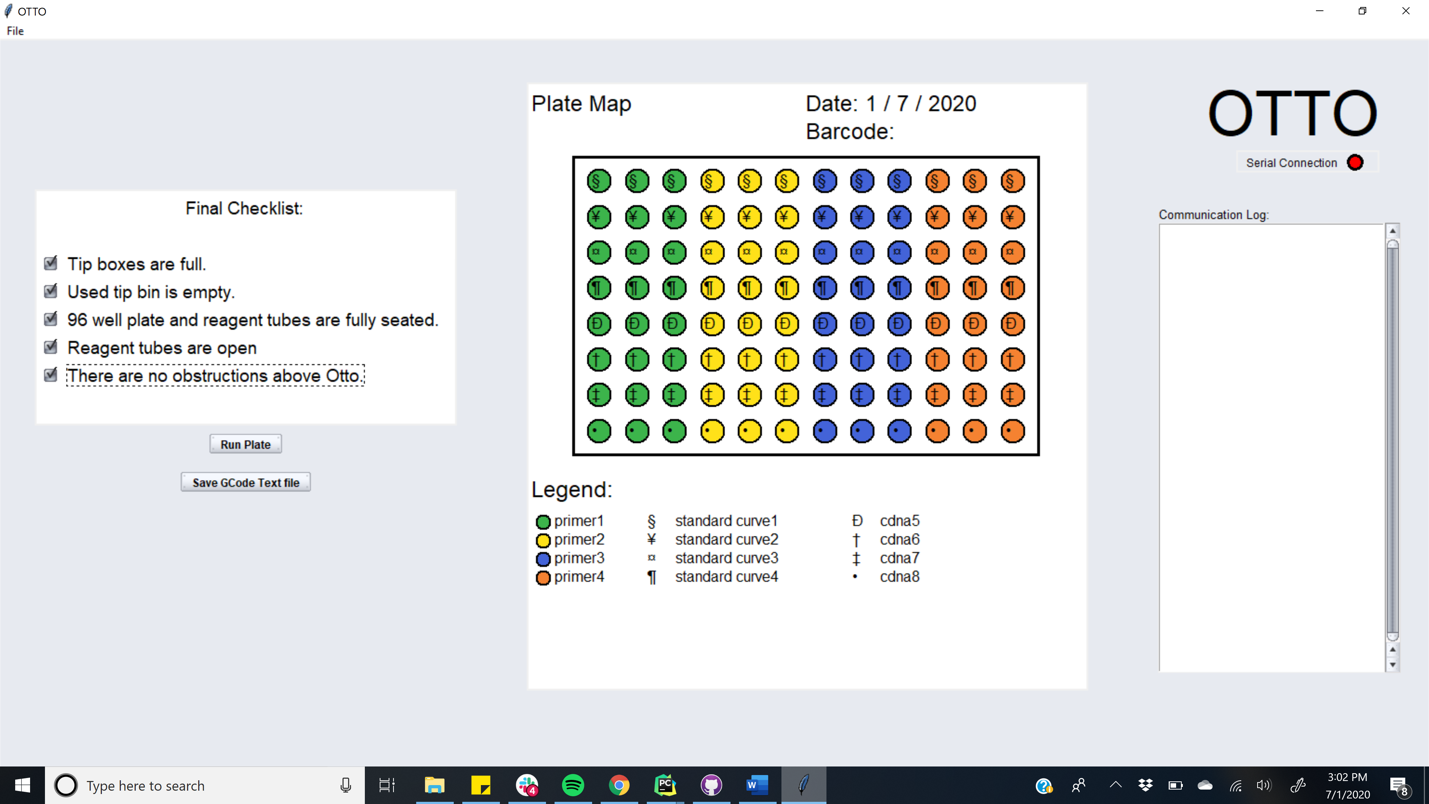


**Supplementary Figure S9.** Run screen. Prior to running the PCR preparation the user must confirm that the tip boxes are full, the tip bin(s) are empty, the 96 well plates and reagent tubes are fully seated, the reagent tubes are uncapped, and there are no obstruction above OTTO. cDNA and primers will be dispensed according to the “Plate Map”, where the colors correspond to the reagent map on the Plate Setup page. Standard curve dilutions and cDNAs are numbered based on their position in the Epi Holder (see Plate Setup page). The user can choose to save the G-code or run the plate from the program.

**Supplementary Table S4.** G-Code interpreter firmware. The Arduino Due is running a custom lightweight firmware that receives and translates G-code generated by Python program and translates these coordinates into motor movements. The Arduino Due can be flashed with this firmware through the [Arduino IDE](https://www.arduino.cc/en/main/software). The firmware requires TMCStepper, AccelStepper, and MultiStepper libraries, which can be installed through the Arduino Library Manager.

| **G-Code Command** | **Purpose** | **Example** |
| --- | --- | --- |
| G1 | Linear Movement in the X-, Y-, Z-, and/or E-axes.  Note: The firmware allows for the pipette actuator to be referred to as the E- or P-axis | G1 X30 Y15 Z20 E5  or  G1 X30 Y15 Z20 P5 |
| G28 | OTTO will home the X, Y, and Z axes. | G28 |
| G38 | Straight probe in the Z direction until floating head switch is triggered. This command is used for picking up tips. | G38 |
| G90 | Absolute positioning.  Note: Currently, the firmware only accepts absolute positional coordinates | G90 |
| M150 | Set LED color.  Note: This is an optional feature. | M150 R20 G50 B100  or  M150 R20 U50 B100 |
| M701 | Load pipette tip.  Note: This is a custom G-code that is similar to the G38 probe routine. | M701 |
| M1001 | Check tip presence and alignment with laser system.  Note: This is a custom G-code and is an optional feature. | M1001 |
