# Supplementary figures and images for: Principles of computer-controlled linear motion applied to an open-source affordable liquid handler for automated micropipetting
Source: Sci Rep. 2020 Aug 12;10:13663. doi: 10.1038/s41598-020-70465-5 (PMC7424513; doi:10.1038/s41598-020-70465-5)

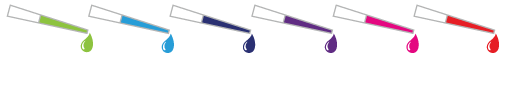

Supplement: Supplementary file 7 — Supplementary Information 6. [file 41598_2020_70465_MOESM7_ESM.zip › pipette_underline.gif]
